# Association of high‐dose radioactive iodine therapy with PPM1D‐mutated clonal hematopoiesis in older individuals

**DOI:** 10.1002/1878-0261.70078

**Published:** 2025-06-26

**Authors:** Jaeryuk Kim, Sungwoo Bae, Jaeyong Choi, Sun‐Wha Im, Bukyoung Cha, Gyeongseo Jung, Sun Wook Cho, Eul‐Ju Seo, Young Ah Lee, Jin Chul Paeng, Young Joo Park, Jong‐Il Kim

**Affiliations:** ^1^ Genomic Medicine Institute, Medical Research Center Seoul National University Seoul Korea; ^2^ Department of Biomedical Sciences Seoul National University Graduate School Seoul Korea; ^3^ Department of Biochemistry and Molecular Biology Seoul National University College of Medicine Seoul Korea; ^4^ Department of Laboratory Medicine, Asan Medical Center University of Ulsan College of Medicine Seoul Korea; ^5^ Institute of Radiation Medicine, Medical Research Center Seoul National University Seoul Korea; ^6^ Department of Biochemistry and Molecular Biology Kangwon National University School of Medicine Gangwon Korea; ^7^ Department of Internal Medicine Seoul National University College of Medicine Seoul Korea; ^8^ Department of Pediatrics Seoul National University Children's Hospital, Seoul National University College of Medicine Seoul Korea; ^9^ Department of Nuclear Medicine Seoul National University Hospital Seoul Korea; ^10^ Department of Molecular Medicine and Biopharmaceutical Sciences, Graduate School of Convergence Science and Technology Seoul National University Seoul Korea; ^11^ Cancer Research Institute Seoul National University Seoul Korea

**Keywords:** error‐corrected next‐generation sequencing, *PPM1D* mutations, radioactive iodine therapy, therapy‐related clonal hematopoiesis, thyroid cancer

## Abstract

While radioactive iodine therapy (RAIT) has been an effective treatment for thyroid cancer, its link to clonal hematopoiesis (CH) has been yet underexplored. In this study, error‐corrected sequencing (median depth: 1926×) of 93 CH‐related genes was performed from the blood samples of 358 thyroid cancer patients, including 110 controls (no RAIT) and 248 RAIT recipients. RAIT recipients were stratified into low‐ and high‐dose groups using a 7.4 GBq cutoff. Multivariable logistic regression revealed that the high‐dose group had a higher CH prevalence with variant allele frequency (VAF) higher than 2% compared to controls, especially in patients aged ≥50 (OR = 2.44, CI = 1.04–6.00, *P* = 0.04). Thirteen genes had mutations with VAF >2%, with *DNMT3A*, *TET2*, and *PPM1D* being the most common. Notably, only the *PPM1D* mutations were significantly linked to RAIT, occurring more frequently in the high‐dose group (13%) compared to the low‐dose group (5%) or controls (2%) at a VAF cutoff of 0.5%. In silico analyses indicated that truncating *PPM1D* mutations confer a selective advantage under high‐dose RAIT and with older age. Although the prognostic implications of *PPM1D*‐mutated CH remain to be further elucidated, these findings offer valuable insights for optimizing RAIT dosing in thyroid cancer patients.

## Graphical abstract

In thyroid cancer patients, high‐dose (≥7.4 GBq) radioactive iodine therapy (RAIT) was associated with a higher prevalence of clonal hematopoiesis (variant allele frequency >2%) in individuals aged ≥50 years (OR = 2.44). *In silico* analyses showed that truncating PPM1D mutations conferred a selective advantage under these conditions. These findings underscore the need to optimize RAIT dosing.

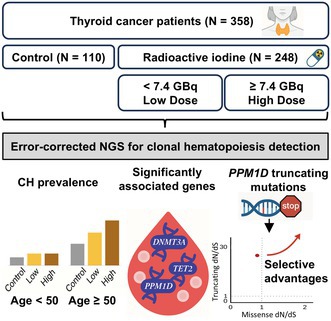

**Abbreviations**
Age_CH_
: age at venipuncture for the CH test
Age_RAIT_
: age at the time of the first RAIT
ARCH‐PD: age‐related clonal hematopoiesis
CH: clonal hematopoiesis
DDR: DNA damage response
dN/dS: nonsynonymous to synonymous substitution ratio
ecNGS: error‐corrected next‐generation sequencing
HSCs: hematopoietic stem cells
NGS: next‐generation sequencing
RAIT: radioactive iodine therapy
t‐MDS: therapy‐related myelodysplastic syndrome
UMI: unique molecular identifier
VAF: variant allele frequencies

## 1 Introduction

Radioactive iodine therapy (RAIT) has been a major treatment modality for thyroid cancer and various benign thyroid diseases [1, 2] with its proven clinical benefits [3, 4, 5]. This therapeutic approach utilizes orally administered radioactive iodine (^131^I), which circulates systemically and selectively accumulates in thyroid tissue, where it emits short‐range beta particles to destroy thyroid follicles [6]. As the global incidence of thyroid cancer has risen over the past three decades [7], the role of RAIT has become increasingly significant.

However, recent studies have raised concerns about its potential side effects, most notably an increased risk of secondary cancers [8, 9, 10, 11, 12, 13]. This risk is not limited to solid cancers but also encompasses hematological malignancies. Several studies have reported a higher incidence of secondary leukemia in patients who have undergone RAIT [8, 13, 14, 15]. Complementing these clinical observations, animal studies using mouse models have demonstrated that radiation exposure can induce dose‐dependent somatic mutations in hematopoietic stem cells (HSCs) [16]. These findings highlight the critical need to investigate RAIT's potential long‐term effects on the hematopoietic system, particularly given its widespread use in thyroid cancer treatment.

The advent of next‐generation sequencing (NGS) technologies has shed light on clonal hematopoiesis (CH), a phenomenon characterized by the expansion of mutated hematopoietic stem and progenitor cells. CH has emerged as a significant risk factor for various health conditions, including blood cancers and cardiovascular diseases [17]. While aging is a well‐established contributor to CH, recent research has revealed that cancer therapies, particularly DNA‐damaging agents, can also induce this condition. Notably, therapy‐related CH often exhibits mutations in DNA damage response (DDR) genes, such as *TP53* and *PPM1D* [18, 19, 20]. The relationship between cancer treatments and CH, however, may vary across therapeutic modalities. While cytotoxic chemotherapy has consistently demonstrated a strong association with CH [18, 19, 20], the impact of RAIT on CH remains less clear. Bolton et al. [19] reported a significant link between radionuclide therapy and CH harboring *PPM1D* mutations; however, their study did not investigate dose‐dependent effects, leaving an important gap in our understanding.

Recent findings suggest that the relationship between DDR gene‐mutated CH and hematopoietic cancers may be more complex than initially thought. While seminal studies indicated that CH with *TP53* mutations is associated with an increased risk of hematologic malignancy [21, 22], a more recent large‐scale cohort study has challenged this notion, showing that neither *TP53* nor *PPM1D* mutations necessarily increase the risk of hematologic cancer—although this study was conducted in a noncancer patient cohort [23]. Meanwhile, in a study involving large nonhematologic cancer cohorts, *TP53* mutations were linked to an increased risk of hematologic malignancy, whereas no such association was found for *PPM1D* mutations [24]. In line with this, Hsu et al. [25] reported no association between prior radiotherapy and CH with *PPM1D* mutations in patients with therapy‐related myelodysplastic syndrome (t‐MDS) or acute myeloid leukemia. Despite these varied findings, a study found that harboring CH in thyroid cancer patients with prior RAIT was associated with decreased survival rates [26]. However, as this study did not conduct gene‐level analyses, the specific genetic factors responsible for this association remain unclear. Therefore, further research is crucial to elucidate the role of different mutations in CH development and progression, as well as potential dose‐dependent relationships between RAIT and CH.

Our retrospective cohort study aimed to investigate the association between RAIT and CH in relation to the administered dosage, with a focus on high‐resolution genetic profiles. To achieve this, we analyzed the mutational profiles of a cohort of thyroid cancer patients stratified by their RAIT history and dosage. Notably, given the growing clinical significance of CH at VAF levels below 2% in various diseases [21, 27, 28], we employed error‐corrected next‐generation sequencing (ecNGS), a technique capable of detecting variant allele frequencies (VAF) below the conventional threshold of 2%, with our VAF cutoff set as low as 0.5%.

## 2 Materials and methods

### 2.1 Study population

Thyroid cancer patients who consented to the use of their biological samples for research purposes at Seoul National University Hospital (SNUH) were recruited following approval by SNUH's Ethical Review Board (IRB No.: 2112‐157‐128). All procedures adhered to the principles of the Declaration of Helsinki. The experiments were undertaken with the understanding and written consent of each subject. Eligible participants were selected after a thorough review of medical records using predefined criteria. Inclusion criteria for selecting participants included: (1) confirmed diagnosis of thyroid cancer between 1981 and 2021; (2) age exceeding 19 at the time they first received RAIT; and (2) a period of more than one year between the first RAIT session and the collection of blood samples if participants had received RAIT. The exclusion criteria included (1) had a prior diagnosis of a malignancy other than thyroid cancer before RAIT initiation and (2) had a history of chemotherapy or radiation therapy before RAIT. Biological samples were collected from June 2011 through October 2022.

Initially, 590 potential participants were identified: 193 individuals had not RAIT at the time of blood collection, while 397 had undergone RAIT. To ensure comparability between the RAIT and control groups, we selected control samples that matched the age distribution of the RAIT group. Additionally, to balance the distribution of sample sizes across RAIT doses, we excluded some samples with doses equal to or lower than 1.11 GBq, resulting in a cohort of 168 controls and 299 RAIT patients. During the sequencing experiment, library preparation failed for samples from 58 controls and 51 RAIT patients. Due to limitations in sample availability, it was not possible to rerun the experiment, leading to the exclusion of these samples from the final analysis. Consequently, our study included 110 controls and 248 RAIT patients (Table 1).

**Table 1:** Demographic and clinical characteristics of subjects. Age_CH_, age at clonal hematopoiesis test; Age_RAIT_, age at the first session of RAIT; ALT, alanine aminotransferase; AST, aspartate aminotransferase; BMI, body mass index; BUN, blood urea nitrogen; CH, clonal hematopoiesis; CRP, C‐reactive protein; ESR, erythrocyte sedimentation rate; FTC, follicular thyroid carcinoma; Hb, hemoglobin; HCT, hematocrit; HDL‐c, high‐density lipoprotein cholesterol; HTN, hypertension; LDL‐c, low‐density lipoprotein cholesterol; MCH, mean corpuscular hemoglobin; MCHC, mean corpuscular hemoglobin concentration; MCV, mean corpuscular volume; MPV, mean platelet volume; PDW, platelet distribution width; PLT, platelet; PTC, papillary thyroid carcinoma; RAIT, radioactive iodine therapy; RBC, red blood cells; RDW, red cell distribution width; T2DM, type 2 diabetes mellitus; VAF, variant allele frequency; WBC, white blood cells.

	Total^b^	Control	Prior RAIT	
			Low (<7.4 GBq)	High (≥7.4 GBq)
Total *N* (%)	358	110 (30.7)	115 (32.1)	133 (37.2)
Age_CH_ (years), mean (SD)	56.2 (14.4)	60.0 (14.5)	52.4 (14.0)	56.4 (13.9)
<50, *n* (%)	116 (32.4)	23 (20.9)	50 (43.5)	43 (32.3)
≥50, *n* (%)	242 (67.6)	87 (79.1)	65 (56.5)	90 (67.7)
Age_RAIT_ (years), mean (SD)	46.4 (13.6)	NA	46.1 (13.2)	46.6 (13.9)
Female sex, *n* (%)	285 (79.6)	94 (85.5)	99 (86.1)	92 (69.2)
Histology				
PTC, *n* (%)	292 (81.6)	78 (70.9)	107 (93.0)	107 (80.5)
FTC, *n* (%)	41 (11.5)	13 (11.8)	6 (5.2)	22 (16.5)
Others^a^, *n* (%)	25 (7.0)	19 (17.3)	2 (1.7)	4 (3.0)
VAF of CH mutations, mean (SD)	1.5 (2.9)	1.3 (1.8)	1.1 (1.0)	2.2 (4.4)
>0.5%, *n* (%)	347 (96.9)	107 (97.3)	113 (98.3)	127 (95.5)
>1%, *n* (%)	117 (32.7)	33 (30.0)	39 (33.9)	45 (33.8)
>2%, *n* (%)	40 (11.2)	11 (10.0)	10 (8.7)	19 (14.3)
>5%, *n* (%)	17 (4.7)	4 (3.6)	1 (0.9)	12 (9.0)
Lymph node or distant metastasis at CH test^b^				
Information not available	161 (44.9)	110 (100.0)	22 (19.1)	29 (21.8)
No, *n* (%)	152 (42.5)	NA	82 (71.3)	70 (52.6)
Yes, *n* (%)	45 (12.6)	NA	11 (9.6)	34 (25.6)
Cumulative RAIT dose (GBq), mean (SD)	9.9 (16.6)	0.0 (0.0)	3.3 (1.7)	23.9 (20.8)
Elapsed month from first RAIT, mean (SD)	97.5 (78.1)	NA	75.9 (74.8)	116.4 (76.3)
Smoking^c^, *n* (%)	339 (94.7)	107 (97.3)	109 (94.8)	123 (92.5)
BMI^d^, mean (SD)	24.5 (3.7)	24.8 (3.6)	24.2 (3.6)	24.5 (3.8)
Medical conditions				
HTN, *n* (%)	123 (34.4)	43 (39.1)	32 (27.8)	48 (36.1)
DM, *n* (%)	60 (16.8)	22 (20.0)	23 (20.0)	15 (11.3)
Dyslipidemia, *n* (%)	13 (3.6)	7 (6.4)	4 (3.5)	2 (1.5)
Hematologic parameters, mean (SD)				
WBC, 10^9^/L	6.6 (3.2)	7.0 (4.6)	6.4 (2.0)	6.3 (2.6)
RBC, 10^12^/L	4.5 (0.5)	4.3 (0.5)	4.6 (0.4)	4.5 (0.5)
Hb, g/dL	13.4 (1.5)	13.1 (1.4)	13.5 (1.4)	13.4 (1.6)
Hct, %	39.9 (4.2)	39.1 (4.3)	40.5 (3.7)	40.2 (4.5)
MCV, fL	89.7 (4.6)	90.7 (4.4)	88.7 (5.1)	89.7 (4.2)
MCH, pg	30.0 (1.8)	30.4 (1.5)	29.7 (2.1)	30.0 (1.8)
MCHC, g/dL	33.4 (1.0)	33.5 (0.9)	33.4 (1.0)	33.4 (1.1)
RDW, %	13.2 (2.1)	13.0 (2.5)	12.9 (1.3)	13.4 (2.1)
PLT, 10^9^/L	249.3 (67.4)	246.2 (77.2)	254.2 (62.8)	247.9 (62.3)
PCT, %	0.2 (0.1)	0.2 (0.1)	0.2 (0.1)	0.2 (0.1)
MPV, fL	9.7 (1.2)	9.6 (1.2)	9.9 (1.1)	9.5 (1.2)
PDW, %	11.9 (2.2)	11.8 (2.0)	12.1 (2.0)	11.8 (2.5)
Biochemical parameters, mean (SD)				
Calcium, mg/dL	8.9 (0.7)	8.9 (0.8)	9.0 (0.6)	8.9 (0.6)
Phosphorus, mg/dL	3.9 (0.7)	3.9 (0.8)	4.1 (0.7)	3.9 (0.6)
BUN, mg/dL	14.1 (4.3)	14.2 (3.9)	13.5 (4.0)	14.6 (4.9)
Creatinine, mg/dL	0.8 (0.2)	0.8 (0.2)	0.8 (0.2)	0.8 (0.2)
AST, IU/L	23.5 (13.7)	26.1 (19.9)	22.5 (8.6)	22.1 (9.9)
ALT, IU/L	23.3 (20.5)	25.3 (29.4)	23.7 (17.7)	21.2 (10.9)
LDL, mg/dL	109.8 (37.7)	114.7 (42.1)	118.6 (40.1)	96.7 (26.9)
HDL, mg/dL	51.1 (12.2)	51.1 (11.7)	54.8 (11.3)	48.1 (13.0)
Total cholesterol, mg/dL	187.8 (39.7)	190.7 (44.8)	188.1 (35.1)	184.9 (38.9)
Inflammatory parameters, mean (SD)				
ESR, mm/h	26.6 (22.9)	29.5 (23.8)	13.2 (7.8)	28.2 (24.4)
CRP, mg/L	3.0 (6.0)	2.8 (4.6)	0.3 (0.4)	4.5 (8.0)

^a^ Other histologic types include anaplastic thyroid cancer (*n* = 15 in the control group, *n* = 1 in the low RAIT group, and *n* = 1 in the high RAIT group), poorly differentiated carcinoma (*n* = 4 in the control group and *n* = 2 in the high RAIT group), and cases with unavailable histologic assessment (*n* = 1 in the low RAIT group and *n* = 1 in the high RAIT group).

^b^ Lymph node or distant metastases at the time of the CH test were assessed using iodine‐131 scans or FDG‐PET; therefore, these evaluations were not possible for the control group, who did not undergo these tests during the initial thyroid cancer assessment.

^c^ Smoking history includes both prior and current smokers.

^d^ The number of patients with available BMI data was 168. Counts for specific laboratory parameters were as follows: WBC, RBC, Hb, HCT, MCH, and MCHC (*n* = 268); MCV, RDW, PLT, PCT, and MPV (*n* = 267); PDW (*n* = 260); calcium and phosphorus (*n* = 281); BUN and AST (*n* = 272); creatinine (*n* = 271); ALT (*n* = 273); LDL (*n* = 57); HDL (*n* = 59); total cholesterol (*n* = 265); ESR (*n* = 73); and CRP (*n* = 82).

RAIT patients were further divided into low‐dose (*n* = 115) and high‐dose (*n* = 133) groups, using a threshold of 7.4 GBq (Fig. S1). Among treated patients, the median cumulative RAIT dose was 7.77 GBq (Fig. S2A). Moreover, analysis to optimize the RAIT dose cutoff for predicting CH (defined as a VAF exceeding 2% or 5%) identified an optimal cutoff of 9.8 GBq (Fig. S2B). However, considering practical and clinical contexts, a cutoff of 7.4 GBq—twice the standard recommended adjuvant dose of 3.7 GBq for remnant ablation after thyroid surgery [5]—was selected.

**Fig. 1:**
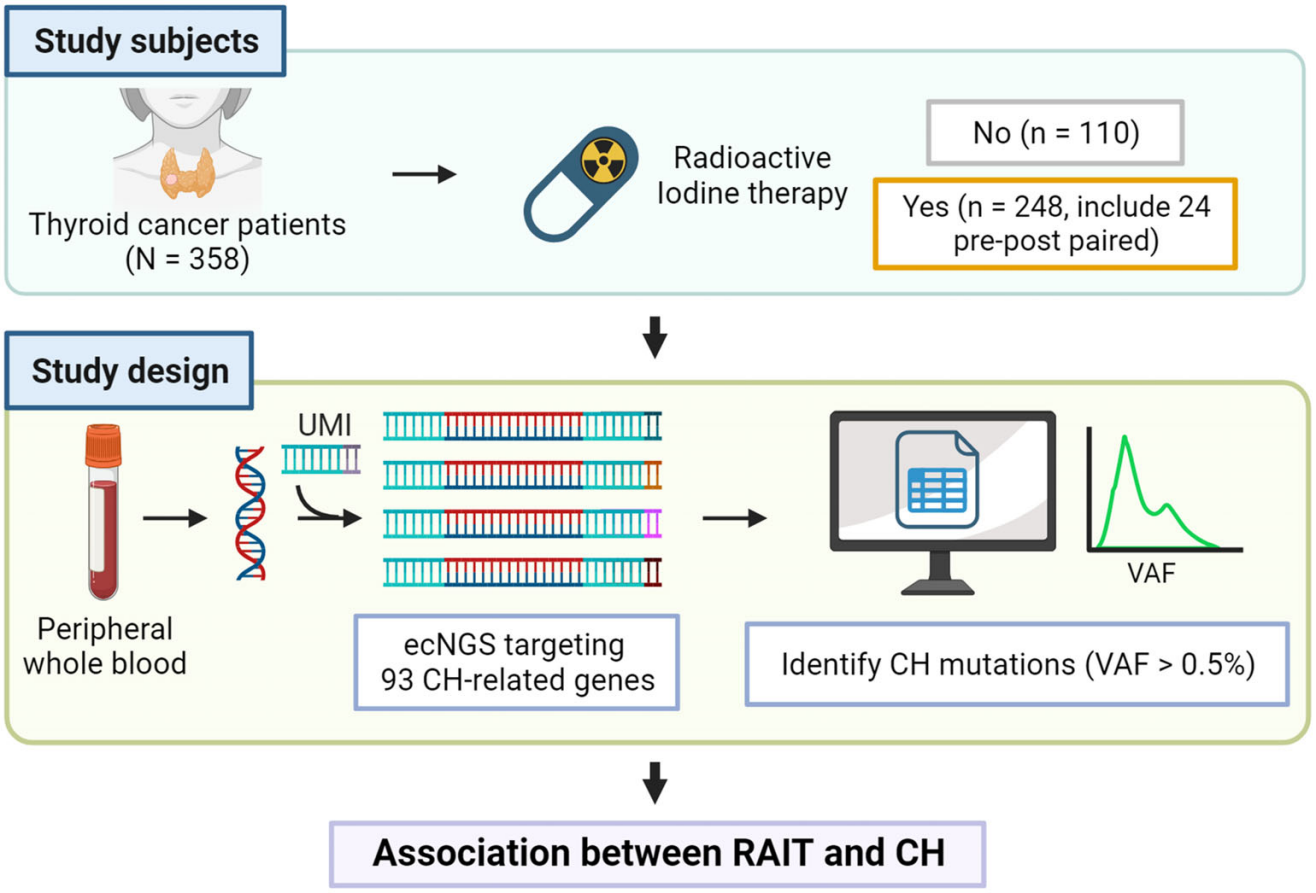
Overview of the study subjects and experimental design. A comprehensive overview of the study. A total of 382 blood samples were collected from 358 patients with thyroid cancer. Among these patients, 110 had no prior history of radioactive iodine therapy (RAIT), while 248 had undergone RAIT, including 24 individuals whose longitudinal samples were collected before and after receiving RAIT. We extracted DNA from the peripheral blood of the patients and conducted error‐corrected next‐generation sequencing (ecNGS) targeting the entire exon regions of 93‐CH‐related genes. We detected mutations with a variant allele frequency (VAF) as low as 0.5%. Consequently, this study aimed to reveal the relationship between the RAIT and CH.

Additionally, pre‐RAIT samples were available for 24 RAIT patients, enabling a longitudinal analysis of CH dynamics following RAIT administration. The overall study population and study design are illustrated in Fig. 1.

### 2.2 Error‐corrected next‐generation sequencing

A custom gene panel targeting exons of 93 genes related to hematologic cancers or CH [19, 29, 30, 31, 32] was designed (Table S1). Using this panel, we sequenced DNA extracted from peripheral blood leukocytes archived in the SNUH Human Biobank. Libraries were constructed with the xGen™ DNA MC Library Prep Kit (IDT), which integrates the xGen UDI‐UMI Adapters (IDT), to enhance the detection of low‐frequency variants. Subsequently, the libraries were sequenced using the NovaSeq 6000 platform (Illumina) at Macrogen (Seoul, South Korea). This approach achieved a median sequencing depth of 1926x after deduplication (Fig. S3). Exploration of the VAF distribution for all identified mutations with a cutoff of 0.5% revealed a distinct right‐skewed pattern (Fig. S4).

### 2.3 Sequencing data processing and variant calling

Raw sequencing reads were aligned to the GRCh37 human genome reference using BWA‐MEM. PCR duplicates were identified and marked based on mapping coordinates. Reads from identical unique molecular identifiers (UMIs) were grouped using fgbio, and consensus reads were aligned to the reference genome. To facilitate the sensitive detection of low‐VAF variants, we used vardict‐java [33], a tool recommended by the manufacturer of the library kit we used. We then applied the stringent criteria to the identified variants to filter potential artifacts and select high‐confidence CH variants.

Additionally, to investigate CH in hotspot regions, we selected putative driver mutations associated with age‐related clonal hematopoiesis (ARCH‐PD) based on the following criteria used in previous studies [21, 34], except for criterion (7), due to insufficient objective evidence for a functionally validated locus. The version of COSMIC data was v101.

1. Truncating mutations in genes implicated in AML pathogenesis by loss‐of‐function (e.g., *NF1*, *DNMT3A*, *TET2*, *IKZF1*, *RAD21*, *WT1*, *KMT2D*, *SH2B3*, *TP53*, *CEBPA*, *ASXL1*, *RUNX1*, *BCOR*, *KDM6A*, *STAG2*, *PHF6*, and *KMT2C*).
2. Truncating variants in *CALR* exon 9.
3. *JAK2*V617F.
4. *FLT3* internal tandem duplication.
5. Nonsynonymous variants at specific hotspot residues (e.g., *CBL*, *DNMT3A*, and *FLT3*).
6. Nonsynonymous variants reported at least 10 times in COSMIC with VAF <42% and a population allele frequency <0.003.
7. Nonsynonymous variants clustering within a functionally validated locus or within four amino acids of a hotspot variant with a population allele frequency <0.003 and VAF <42%.
8. Nonsynonymous variants reported in COSMIC >100 times with a population allele frequency <0.003 regardless of VAF.

### 2.4 Variant filtering strategies

We subsequently filtered variants using the following strategies.

1. Exclusion of common sequencing artifacts: Variants susceptible to common sequencing artifacts, such as orientation bias and strand bias, were filtered out using sequence orientation bias (SOB) detector [35] and the built‐in code in the vardict‐java tool.
2. Germline variant exclusion: Variants with a population allele frequency >1% in either the gnomAD whole‐exome or gnomAD whole‐genome database were excluded because they were likely to be germline in origin. Additionally, variants with a VAF exceeding 35% were filtered out, as they were also considered germline in origin. In addition, a manual inspection of variants exceeding a VAF of 20% was performed with the dbSNP database to further filter out rare germline variants.
3. Exclusion of error‐prone regions: Regions known for their susceptibility to sequencing errors were excluded from the analysis. This was achieved by incorporating genome stratification files developed by the Global Alliance for Genomic Health Benchmarking Team, the Genome in a Bottle Consortium, and the Telomere‐to‐Telomere Consortium [36]. Specifically, we masked genomic regions characterized by low complexity, encompassing tandem repeats and homopolymer regions, as well as their flanking 5 bp regions.
4. Exclusion of low‐confidence variants: Only variants meeting stringent depth and count criteria were retained for further analysis. Specifically, variants with a sequencing depth exceeding 400× and an alternative read count >2 for single nucleotide polymorphisms and >5 for insertion–deletion mutations were included. Furthermore, variants occurring multiple times in more than 10% of the total samples were excluded because they were deemed likely attributable to technical errors.

The remaining filtered variants were subjected to comprehensive annotation using two annotation tools, Funcotator and ANNOVAR. For downstream analyses, we exclusively considered nonsynonymous variants except for nonsynonymous to synonymous (dN/dS) ratio analysis, which requires synonymous variant information.

### 2.5 *In silico* variant pathogenicity prediction

To explore the pathogenicity of CH mutations, we utilized variant pathogenicity prediction scores of various *in silico* prediction tools offered by ANNOVAR. Specifically, we assessed scores generated by 11 commonly employed prediction tools that have differing algorithms: CADD [37], DANN [38], FATHMM‐MKL [39], SIFT [40], MutationAssessor [41], PrimateAI [42], PROVEAN [43], MetaLR [44], MetaSVM, M‐CAP [45], and REVEL [46].

### 2.6 Assessing selective advantage using dN/dS analysis

To investigate selection advantages in the context of RAIT, we conducted a dN/dS analysis. For this analysis, we utilized the dNdScv R package [47], a specialized tool for analyzing maximum likelihood dN/dS ratios tailored to assess selection in specific scenarios. The analysis was conducted using the default package parameters. Genes exhibiting significant selection were identified, with a threshold *q*‐value of <0.10 considered significant. A dN/dS ratio <1 indicates negative selection, that equal to 1 represents neutral selection, and that >1 denotes positive selection.

### 2.7 Statistical analysis

Statistical analyses were performed using R, with significance set at <0.05. Advanced R packages, including rstatix (https://github.com/kassambara/rstatix), finalfit (https://github.com/ewenharrison/finalfit), and sjPlot (https://github.com/strengejacke/sjPlot), were used. Firth's logistic regression, implemented through the logistf package (https://github.com/cran/logistf), used for multiple logistic regression with sparse data.

## 3 Results

### 3.1 Clonal hematopoiesis is dependent on both patient age and radioactive iodine dose

**Fig. 2:**
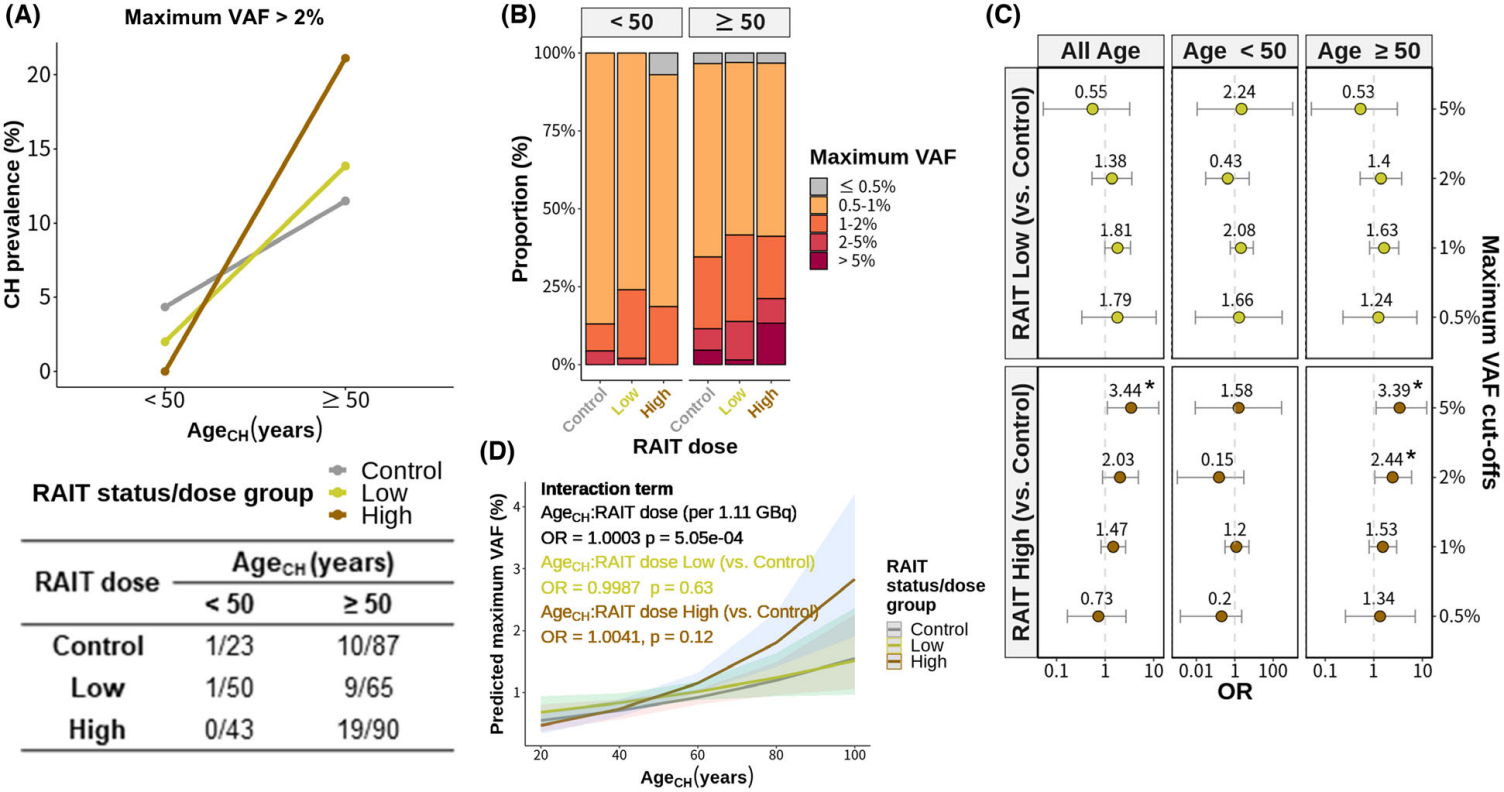
Associations between age, radioactive iodine therapy dose, and clonal hematopoiesis. (A) The prevalence of clonal hematopoiesis (CH), defined by a variant allele frequency (VAF) exceeding 2%, was notably greater in older patients (age at CH test; Age_CH_ ≥ 50) across all three groups: control (*n* = 110), low‐dose (*n* = 115), high‐dose (*N* = 133). (B) Distribution of the maximum VAF in each individual according to Age_CH_ and radioactive iodine therapy (RAIT) status/dose group. Mutations with high‐VAF levels were more prevalent at older ages (Age_CH_ ≥ 50 years), particularly within the high‐dose RAIT group. (C) In multivariable logistic regression analyses for the presence of CH with varying VAF cutoffs adjusting for Age_CH_, sex, and smoking status, the high‐dose RAIT group displayed a significant association compared to the control group for 2% and 5% VAF cutoffs, but only in older patients (Age_CH_ ≥ 50 years). Error bars indicate confidence interval. (D) A plot of the marginal effect model with an interaction term between Age_CH_ and RAIT status/dose for maximum VAF. Although there was no significant difference between the RAIT‐received groups and the control group (colored lines and text), a significant interaction emerged when a continuous RAIT dose was included as an independent variable (black text) (Table S3). The maximum VAF is defined as the VAF of the mutation with the highest frequency in an individual. In regression analyses, the VAF was log‐transformed. Firth logistic regression was used to test for the presence of CH because of the sparse data. The units of the Age_CH_ and RAIT doses are per 10 years and per 1.11 GBq increase, respectively. *P value <0.05.

First, we explored the association between age and CH across RAIT status/dose groups. The prevalence of CH, defined as a VAF exceeding 2%, notably increased with patient age across all three groups, which was evident when age at venipuncture for the CH test (Age_CH_) was categorized into two groups (younger than and older than or equal to 50 years) (Fig. 2A) or by decades (Fig. S5A).

Subsequently, we examined the distribution of the maximum VAF for CH mutations in each sample, considering both age and administered RAIT dose (Fig. 2B). A substantial increase in the proportion of individuals with a maximum VAF exceeding 5% was noted in older age groups (Age_CH_ ≥ 50 years), especially among those exposed to higher RAIT doses (Table S2). This trend persisted even when patients were stratified by Age_CH_ into decades (Fig. S5B). In addition, even when focusing only on hotspot mutations corresponding to ARCH‐PD, CH prevalence increased with age (Fig. S6A), although in individuals under 40 the prevalence of CH below 1% was higher than in older patients (Fig. S6B).

Next, we investigated the number of CH mutations across various VAF cutoff points (Fig. S7). For cutoff points of 0.5%, 1%, and 2%, CH mutation counts per sample did not differ across RAIT status/dose groups. However, a statistically significant higher number of mutations emerged for a VAF cutoff of 5%, but only in older age groups (Age_CH_ ≥ 50 years) within the high‐dose RAIT group.

To elucidate the factors influencing the clone size of CH quantified by the VAF, we performed linear regression analyses on the Age_CH_ and RAIT doses using log‐transformed VAF data, taking into account the skewed distribution of the VAF. As expected, a significant linear relationship emerged between Age_CH_ and VAF (Fig. S8A). Remarkably, a significant linear relationship between the RAIT dose and VAF was also identified (Fig. S8B). However, in both cases, the correlation coefficients were <0.4, indicating a weak positive correlation.

Then, multivariable linear regression analyses incorporating age and RAIT dose variables, the latter being both categorical and continuous, were conducted (Fig. S9). The findings indicated a significant association between higher RAIT doses and elevated VAF. Notably, when Age_CH_ were stratified based on a cutoff of 50 years, only older patients exhibited a significant association (Fig. S9A). Among RAIT‐treated patients, a similar trend was observed when age at the time of the first RAIT (Age_RAIT_) was used instead of Age_CH_ and the elapsed time since the first RAIT was included as an independent variable (Fig. S9B).

Furthermore, when multivariable logistic regression analyses assessing the presence of CH with varying VAF cutoffs were performed, significant associations were found only for high‐dose RAIT, particularly among the older age group (Age_CH_ ≥ 50 years) (Fig. 2C; Fig. S10): for a maximum VAF cutoff of 0.5%, OR = 1.34 (CI = 0.26–7.11, *P* = ns); 1%, OR = 1.53 (CI = 0.80–2.95, *P* = ns); 2%, OR = 2.44 (CI = 1.05–6.00, *P* < 0.05); and 5%, OR = 3.39 (CI = 1.11–12.28, *P* < 0.05).

To further explore the interaction between Age_CH_ and the RAIT dose, a multivariable linear regression analysis introducing an interaction term between these factors was conducted (Fig. 2D; Table S3). When the RAIT status/dose group was included as a categorical variable, no significant interaction effect was observed between the RAIT‐received groups and the control group. However, when the RAIT dose was treated as a continuous variable, a significant interaction effect was identified.

Taken together, these results suggest increased susceptibility to CH development following high‐dose RAIT, particularly in older individuals.

### 3.2 Clonal hematopoiesis with specific genetic alterations is associated with high‐dose radioactive iodine therapy

**Fig. 3:**
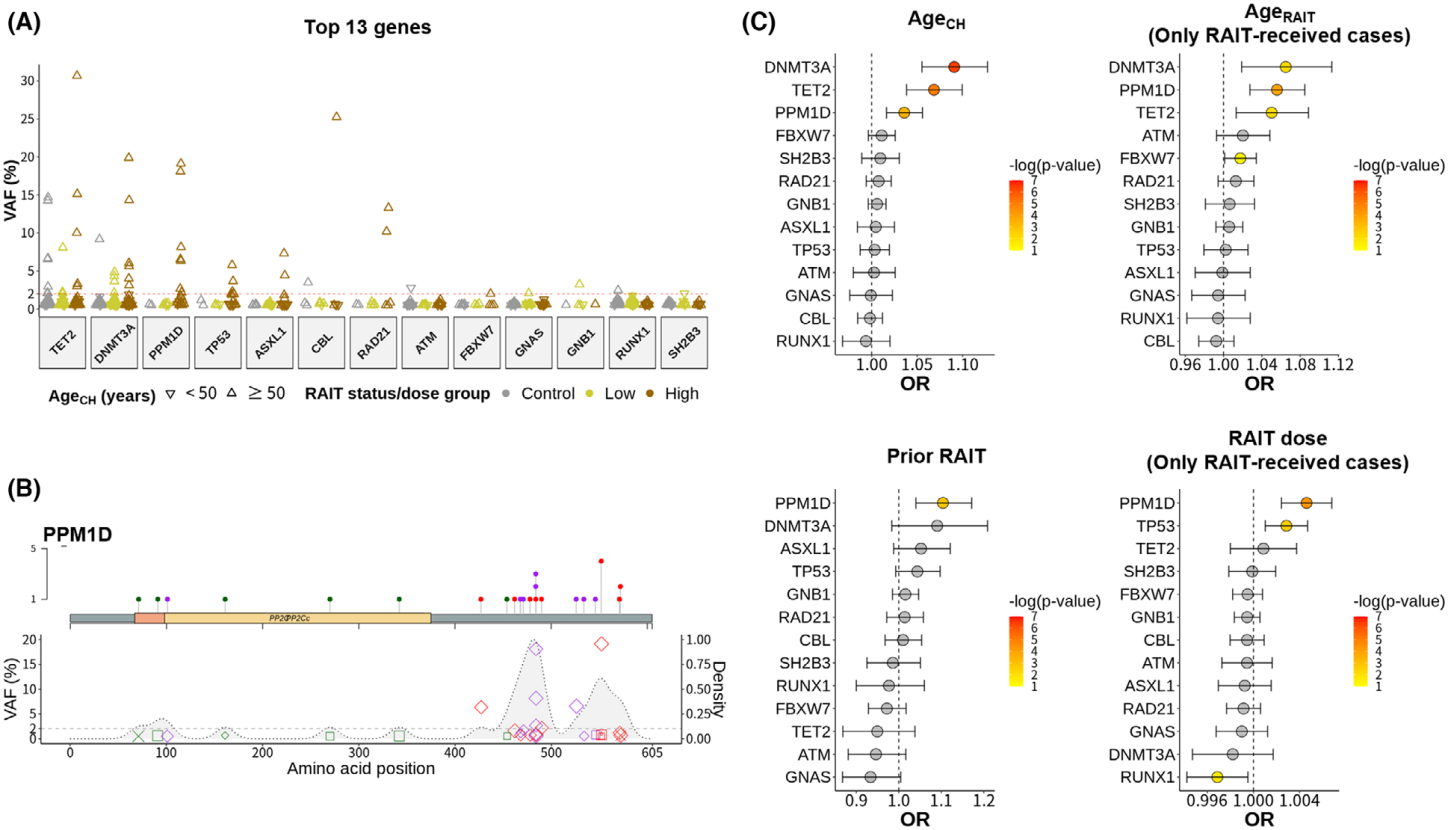
Association of high‐dose radioactive iodine therapy with *PPM1D*‐mutated clonal hematopoiesis. (A) Analysis of mutations in the top 13 genes, chosen for having variant allele frequency (VAF) exceeding 2% in at least one sample, concerning age at clonal hematopoiesis test (Age_CH_) and radioactive iodine therapy (RAIT) status/dose. High‐VAF mutations were notably more prevalent in older patients, and certain genes (*PPM1D, TP53, ASXL1, RAD21*) showed enrichment specifically in the high‐dose RAIT group. Sample sizes: Control (*n* = 110), low‐dose RAIT (*n* = 115), and high‐dose RAIT (*n* = 133). (B) Lollipop plots highlighting mutation positions and frequencies in *PPM1D* with the VAF of corresponding positions and their frequency densities (gray background). High‐VAF truncating mutations in *PPM1D* clustered near the C terminus, setting them apart from mutations in other genes. (C) Multivariable logistic regression analysis of the associations between the presence of CH mutations in the top 13 genes and multiple clinical variables. Analyses were conducted using mutation presence (VAF >0.5%) in each gene as the dependent variable. The independent variables included Age_CH_ (adjusted for sex, smoking, prior RAIT), age at first RAIT (Age_RAIT_; adjusted for sex, smoking, RAIT dose, elapsed time from first RAIT), prior RAIT (adjusted for Age_CH_, sex, smoking), and RAIT dose (adjusted for Age_CH_, sex, smoking, elapsed time from first RAIT). Nonsignificant results (*P* value >0.05) are presented as gray dots. Error bars indicate confidence interval.

We further investigated the association between high‐dose RAIT and CH by examining mutations at the gene level. We focused on the top 13 genes identified with a VAF exceeding 2% in at least one sample (Fig. 3A; Table S4). As a result, mutations in the well‐established age‐associated CH genes, *TET2* and *DNMT3A* were most frequent. Notably, high‐VAF (>2%) mutations exhibited a greater prevalence in older patients (Age_CH_ ≥ 50 years), in genes such as *TET2*, *DNMT3A*, *PPM1D*, *TP53*, and *ASXL1*. When only the mutation with the maximum VAF per individual was considered, similar results were obtained (Table S5).

To further elucidate the distribution of CH mutations with respect to Age_CH_ and RAIT dose, we generated oncoplots for the top 13 genes at various VAF cutoff values (Fig. S11). In particular, *PPM1D* mutations exhibited prominent enrichment in high‐dose RAIT at all VAF cutoffs, displaying a distinct pattern characterized by the enrichment of truncating mutations, specifically nonsense and frameshift mutations.

Next, we generated lollipop plots to illustrate the mutation positions and frequencies of high‐VAF genes, specifically *TET2*, *DNMT3A*, *PPM1D*, and *TP53* (Fig. 3B; Fig. S12). Overall, CH mutations tended to cluster within functional domains, while *PPM1D* exhibited clustering of truncating mutations near the C terminus associated with polyubiquitination sites for protein degradation [48].

To determine the effect of each gene mutation, multivariable logistic regression analyses were conducted (Fig. 3C). The dependent variable was the presence of mutations in each gene with a VAF cut‐off of 0.5%, and the independent variables included Age_CH_, Age_RAIT_, prior RAIT, and RAIT dose and were adjusted for sex, smoking status, and for patients who received RAIT, the time elapsed since the first RAIT was also included. We revealed that, in addition to *TET2* and *DNMT3A* mutations, *PPM1D* mutations were positively associated with both Age_CH_ and Age_RAIT_. Moreover, only *PPM1D* mutations exhibited a positive association with prior RAIT. Additionally, *TP53* and *PPM1D* mutations were positively associated with the administered RAIT dose. Extending our analyses to genes beyond the top 13, we did not observe significant positive associations, except for *GATA2* with prior RAIT and *ZRSR2* with RAIT dose (Fig. S13).

To further investigate the association of DDR genes (*PPM1D* and *TP53*) with RAIT status/dose, we conducted multivariable logistic regression analyses focusing on these genes at various VAF cutoffs. We found that in patients aged ≥50, the odds ratio for high‐dose RAIT was considerably higher than that observed when all CH was considered (Fig. S14): for a maximum VAF cutoff of 0.5%, OR = 8.71 (CI = 1.85–84.9, *P* < 0.01); for 1%, OR = 6.39 (CI = 1.28–63.70, *P* < 0.05); for 2%, OR = 15.22 (CI = 1.54–2.07 × 10^3^, *P* < 0.05); and for 5%, OR = 20.70 (CI = 8.26–2.99 × 10^4^, *P* = ns). In contrast, analyses for DTA genes (*DNMT3A*, *TET2*, and *ASXL1*)—which are well known to be age‐related—revealed no significant association with RAIT status/dose, regardless of age (Fig. S15).

Collectively, our findings indicate a significant association between specific genetic alterations, particularly *PPM1D* mutations, and CH in the context of older age and high‐dose RAIT.

### 3.3 Certain clonal hematopoiesis mutations are associated with selective advantages in high‐dose radioactive iodine therapy

**Fig. 4:**
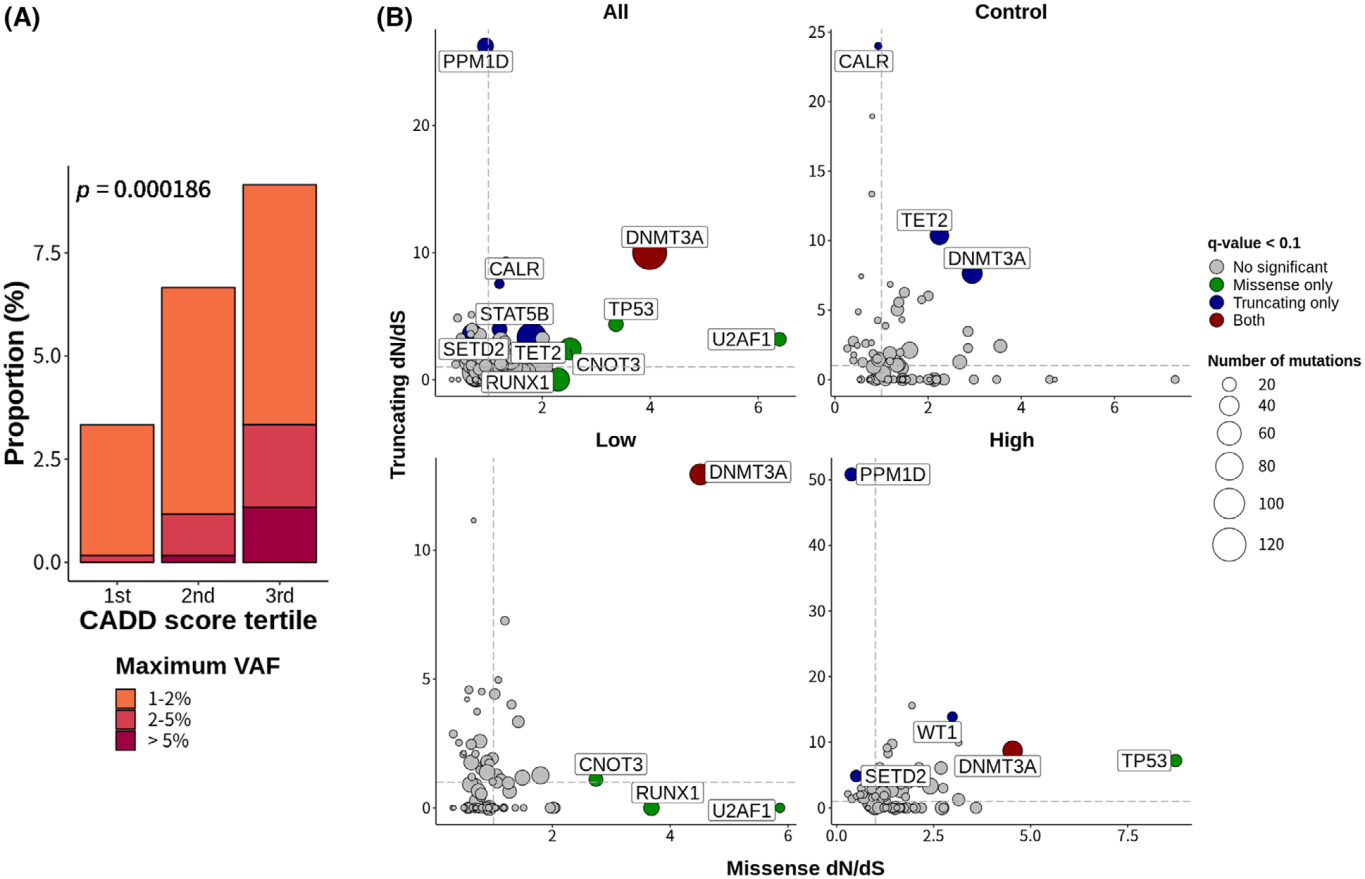
Association between functional alterations in clonal hematopoiesis mutations and positive clonal selection. (A) Clonal hematopoiesis (CH) mutations were categorized according to the tertiles of CADD scores, and the proportion of clone sizes, determined by variant allele frequency (VAF) in each tertile, was analyzed. A trend was observed indicating a greater VAF with higher CADD scores. (B) Analysis of the nonsynonymous to synonymous substitution (dN/dS) ratio of each gene in all individuals and within each radioactive iodine therapy (RAIT) status/dose group revealed positive selection (dN/dS >1) for truncating *PPM1D* and missense *TP53* mutations, particularly in the high‐dose RAIT group. The vertical and horizontal gray dashed lines indicate a dN/dS ratio of 1.

To obtain a deeper understanding of the selective fitness advantages exhibited by specific clones in relation to RAIT, we hypothesized that CH clone size would be related to functional alterations. In this regard, we investigated the association of the VAF, a representative measure of clone size, with CADD scores, which predict *in silico* mutation pathogenicity. We found that mutations with higher CADD scores were associated with a greater VAF, as shown by analysis of CADD score tertiles (Fig. 4A) and linear regression (Fig. S16A). This result suggested that certain types of functional disruption are associated with the positive selection of affected clones. This correlation was further supported by other *in silico* pathogenicity prediction tools (Fig. S16B–K). Specifically, eight out of the 10 tools with distinct prediction algorithms showed significant relationships between pathogenicity scores and VAF.

Additionally, leveraging the fact that we sequenced all the exon regions of the targeted genes, we examined the ratio of nonsynonymous to synonymous substitutions (dN/dS), an indicator of clonal selection within each gene across the RAIT status/dose groups (Fig. 4B). Notably, we observed that truncating *PPM1D* mutations and missense *TP53* mutations exhibited dN/dS ratios >1 exclusively in the high‐dose RAIT group, indicating positive selection of these genes. Furthermore, when subgrouping by age of 50 years, only older patients (≥50 years) displayed positive selection of specific genes, including *DNMT3A*, *TET2*, and *PPM1D* (Fig. S17).

Next, to gain clues about the functional relationships between the affected genes or pathways, we investigated the mutual exclusiveness or co‐occurrence of the detected mutations with various VAF cutoffs (Fig. S18). We detected a co‐occurrence of *PPM1D* and *TP53* mutations when the VAF cutoff was 1%, aligning with a previous study indicating a significant co‐occurrence of *PPM1D* and *TP53* mutations in t‐MDS [49].

Taken together, our results suggest that functional alterations in specific genes or interactions between affected clones may play a crucial role in influencing the selective advantages of certain CH clones, particularly in the context of high‐dose RAIT.

### 3.4 Longitudinal analysis of hematopoietic clonal dynamics following low‐dose radioactive iodine therapy

**Fig. 5:**
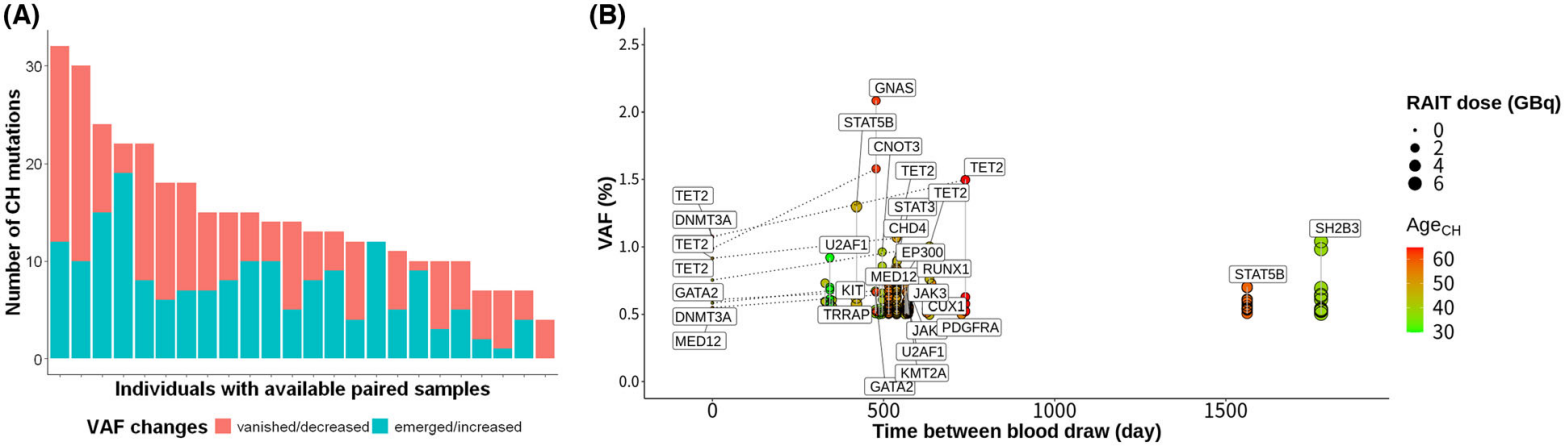
Longitudinal assessment of clonal hematopoiesis before and after the radioactive iodine therapy. (A) The figure presents the number of CH mutations that changed the variant allele frequency (VAF) before and after radioactive iodine therapy (RAIT) for 24 patients with paired blood samples. Mutations undetectable after RAIT or those with decreased VAF were classified as ‘vanished/decreased’, while new mutations or those with increased VAF were classified as ‘emerged/increased’. (B) VAF changes for paired samples were examined based on the time between venipunctures. The figure exclusively displays existing mutations with elevated VAF or newly acquired mutations after RAIT. All these samples were obtained from individuals who received low‐dose RAIT (<7.4 GBq). The gray vertical lines connect all mutations detected in a sample, and the gray dashed lines connect mutations detected both before and after RAIT. Gene names are selectively labeled for mutations both detected before and after RAIT or for mutations with a maximum VAF after RAIT.

To unravel the dynamic changes in the clonal composition of CH influenced by RAIT, we conducted a longitudinal analysis of clone size changes in 24 individuals with available blood samples both before and after RAIT (Fig. 5; Table S6). First, we examined the change in the number of CH mutations pre‐ and post‐RAIT (Fig. 5A). Mutations undetectable after RAIT or those with decreased VAF were classified as ‘vanished/decreased’, while new mutations or those with increased VAF were classified as ‘emerged/increased’. Interestingly, the average number of vanished/decreased and emerged/increased mutations per patient was comparable (7.7 vs. 7.8).

Subsequently, we investigated the changes in the VAF over time between blood draws, specifically for mutations of emerged/increased (Fig. 5B). Overall, we did not observe abrupt increases in the VAF or emergence of high‐VAF mutations, except in some older patients. Moreover, the emerged/increased mutations were not associated with those typically implicated in high‐dose RAIT, such as truncating *PPM1D* mutations.

Due to sample limitations that all the samples were obtained from low‐dose RAIT patients, investigations of clonal dynamics during high‐dose RAIT were limited. Nevertheless, our findings suggest that under low‐dose RAIT conditions, hematopoietic clonal populations demonstrate minimal positive selection advantages.

## 4 Discussion

Our study uncovered an association between RAIT and CH in relation to dosage and aging. We also found that clones with specific genetic alterations, particularly truncating *PPM1D* mutations, gained fitness advantages in response to high‐dose RAIT. Methodologically, we employed ecNGS, which comprehensively targeted entire exon regions with VAF sensitivity as low as 0.5%. This approach enabled in silico analyses that uncovered selective fitness advantages in clones with specific mutations under certain conditions—an analysis that was not feasible in previous studies that focused solely on mutation hotspots [29, 31] and/or used a higher VAF threshold of 2% [19, 26].

Various environmental stressors drive mutation‐specific CH [50]. While aging is commonly associated with *DNMT3A* and *TET2* mutations, genotoxic stressors are linked to mutations in DDR genes such as *TP53* and *PPM1D*. Under genotoxic stress, pre‐existing clones containing DDR gene mutations are thought to become resistant to therapy and gain survival advantages, leading to clonal expansion [51, 52]. In this study, we found that specific genes, including *PPM1D* and *TP53*, were prevalent in patients who received high‐dose RAIT. Notably, *PPM1D*, a key negative regulator of the p53 pathway [25, 48, 53], was characterized by truncating mutations that confer gain‐of‐function effects. Additionally, dN/dS ratio analysis indicated positive clonal selection for truncating *PPM1D* mutations and missense *TP53* mutations under high‐dose RAIT. Supporting this, previous studies using a bone marrow transplantation mouse model demonstrated that clones harboring truncating *PPM1D* mutations [54, 55] or missense *TP53* mutations [54] exhibited significant competitive fitness advantages over the wild‐type, particularly in the presence of genotoxic stress. In line with this experimental evidence, our results suggest that RAIT acts as a genotoxic stressor, conferring selective advantages for clones with DDR gene mutations.

Emerging evidence suggests that *PPM1D*‐mutated CH is associated with significant clinical outcomes. For instance, individuals with *PPM1D*‐mutated CH prior to autologous stem cell transplantation exhibited inferior overall survival compared to patients with other mutations [56]. Similarly, pre‐existing *PPM1D*‐mutated CH was associated with worse overall survival in patients with nonsmall cell lung cancer who underwent surgical resection followed by adjuvant therapy [52]. Although experimental evidence indicates that PPM1D mutations confer resistance to cancer therapy in hematopoietic cells [25, 48, 53, 54, 55], large studies have demonstrated that *PPM1D*‐mutated CH is not clearly associated with an increased risk of hematologic malignancies in both noncancer [23] and nonhematologic cancer [24] cohorts. Additionally, in AML or MDS patients, *PPM1D*‐mutated clones often spontaneously regressed following discontinuation of DNA‐damaging therapy and only rarely dominated as founding clones [57]. These findings collectively suggest that the potentially poorer prognosis associated with *PPM1D*‐mutated CH may primarily result from nonhematological complications, such as exacerbated heart failure [58] and immune system alterations [59], rather than progression to hematologic malignancy. However, further investigation into the exact clinical significance and biological roles of *PPM1D*‐mutated CH is warranted.

Given the potential prognostic significance of *PPM1D*‐mutated CH, our findings linking high‐dose RAIT with this mutation highlight the importance of precisely determining RAIT doses. However, it is important to note that our study did not observe a notable emergence of new clones or an increase in VAF after more than one year following low‐dose RAIT (<7.4 GBq). This aligns with earlier findings showing no significant change in the VAF of CH over two years following adjuvant therapeutic doses of RAIT [60]. Therefore, we believe that RAIT administered at remnant ablation and adjuvant doses below 3.7 GBq, RAIT remains a viable option, given its significant clinical benefits and minimal associated risks.

We demonstrated an age‐dependent increase in both the size of CH clones and the overall prevalence of CH, which aligns with the well‐established concept that aging is related to the development of CH. Furthermore, multivariable regression analyses that incorporated the interaction between age and RAIT dose highlighted the synergistic effects of these factors on the extent of CH. As HSCs age, they gradually lose their self‐renewal capacity due to cumulative DNA damage, cellular senescence, impaired autophagy and mitochondrial function, and epigenetic reprogramming [61]. In this compromised environment, pre‐existing mutations in DDR genes can confer a competitive advantage, enabling these clones to better withstand further DNA damage induced by genotoxic stress, such as radiation. While our findings support this theoretical explanation, further experimental studies are needed to unravel the complex interactions between genotoxic stress and aging in CH.

In our study, we included variables known to be associated with the development of CH—such as age, smoking history, and prior cancer therapy—in our regression analyses. However, we cannot entirely rule out the possibility that tumor factors (e.g., histologic type and stage) or other host factors (e.g., germline predisposition [62]) may also influence CH development. Indeed, when we limited our analysis to the hotspot region defined by ARCH‐PD, we observed that patients younger than 40 exhibited a higher prevalence of CH than those in their 40s (Fig. S6B), suggesting a possible link to germline predisposition that may also contribute to the earlier onset of thyroid cancer in these patients. Alternatively, the inherent characteristics of early‐onset thyroid cancer might influence CH development; however, there is currently insufficient evidence to support a direct impact of cancer on CH occurrence. Meanwhile, several studies suggest that CH may influence the progression of solid tumors and be associated with adverse outcomes; for example, one study reported that the VAF of CH infiltrating the tumor microenvironment in anaplastic thyroid cancer is higher than that in peripheral blood [63]. While these findings imply that CH might affect the development or progression of solid tumors, future experimental studies are warranted to further investigate the potential interactions between cancer and CH.

Recent studies suggest that CH with variant allele frequencies VAF below 2% is common in healthy individuals [31, 64]. Although the clinical significance of these low‐VAF CH cases remains to be fully elucidated, emerging evidence indicates its potential relevance in various diseases, particularly in cardiovascular risk assessment [28] and hematologic malignancy prediction [21]. In this context, the RAIT‐associated CH observed in our study—particularly involving *PPM1D* mutations with VAFs above 0.5%—underscores the importance of further investigation into the biological relevance and long‐term outcomes associated with these low‐VAF mutations. In addition, larger cohort studies focusing on CH cases with VAFs above 2%—where clinical relevance is more established—are needed to confirm the impact of RAIT on CH.

## 5 Conclusions

In summary, our study established a dose‐dependent association between RAIT and CH, particularly in older individuals. Using ecNGS, we achieved highly sensitive detection of mutant clones and found that specific genetic alterations, most notably truncating *PPM1D* mutations, undergo positive clonal selection following high‐dose RAIT. Given the potential prognostic implications of *PPM1D*‐mutated CH, our findings may offer valuable insights for refining treatment strategies and ultimately enhancing the long‐term health outcomes of thyroid cancer survivors receiving RAIT.

## Conflict of interest

The authors declare no conflict of interest.

## Author contributions

JK performed study design, data analysis, and drafted the research protocol for IRB approval, and wrote the manuscript. JIK supervised the study design and provided guidance on analysis direction. YJP supervised the study design, sample collection, clinical information research, and analysis direction and contributed to manuscript writing. JCP contributed to study design, analysis direction, and contributed to writing the research protocol for IRB approval. SB contributed to analysis direction and the investigation of patients' clinical information. YAL provided expertise in study design and analysis direction. EJS contributed to enhancing the quality of analysis and manuscript writing. BC conducted the sequencing experiments. SWI and JC contributed sequencing data analysis. GJ contributed to sample collection and preparation. SWC contributed to sample collection and clinical information research.

## Peer review

The peer review history for this article is available at https://www.webofscience.com/api/gateway/wos/peer‐review/10.1002/1878‐0261.70078.

## Supporting information


**Fig. S1.** Distribution of radioactive iodine therapy dose in our study cohort.
**Fig. S2.** Determination of radioactive iodine therapy dose cut‐off for low and high groups.
**Fig. S3.** Distribution of coverage depth for detected clonal hematopoiesis mutations.
**Fig. S4.** Distribution of the variant allele frequency of detected all clonal hematopoiesis mutations.
**Fig. S5.** Association of age and radioactive iodine therapy status/dose with clonal hematopoiesis.
**Fig. S6.** Prevalence of putative driver mutations associated with age‐related clonal hematopoiesis (ARCH‐PD) at various variant allele frequency thresholds.
**Fig. S7.** The number of clonal hematopoiesis mutations per individual across radioactive iodine therapy status/dose groups at various variant allele frequency cutoffs.
**Fig. S8.** Association of age and radioactive iodine therapy dose with variant allele frequency of clonal hematopoiesis.
**Fig. S9.** Multivariable linear regression analyses for maximum variant allele frequency.
**Fig. S10.** Multivariable logistic analyses examining the presence clonal hematopoiesis with various variant allele frequency cut‐offs.
**Fig. S11.** Oncoplots illustrating CH mutations in the top 13 genes at various variant allele frequency cut‐offs.
**Fig. S12.** Lollipop plots highlighting mutation positions and frequencies in *TET2, DNMT3A, TP53* genes.
**Fig. S13.** Multivariable logistic regression analysis for the association of clonal hematopoiesis mutations in each gene (excluding the top 13 genes) with clinical variables.
**Fig. S14.** Multivariable logistic analyses for the presence of DNA damage response genes (*PPM1D* and *TP53*) mutated clonal hematopoiesis with various variant allele frequency cut‐offs.
**Fig. S15.** Multivariable logistic analyses for the presence of DTA genes (*DNMT3A, TET2, and ASXL1*) mutated clonal hematopoiesis with various variant allele frequency cut‐offs.
**Fig. S16.** Relationship between variant allele frequency of clonal hematopoiesis mutations and predicted pathogenicity scores.
**Fig. S17.** Analysis of nonsynonymous to synonymous substitution ratio (dN/dS) in clonal hematopoiesis‐related genes across age groups.
**Fig. S18.** Mutual exclusivity and co‐occurrence of genes in CH with various VAF cut‐offs.
**Table S1.** A list of clonal hematopoiesis‐related genes targeted in this study.
**Table S2.** Patient characteristics harboring clonal hematopoiesis mutations with variant allele frequency exceeding 5%.
**Table S3.** Interaction terms between age at clonal hematopoiesis test (AgeCH) and radioactive iodine therapy dose or status/dose group.
**Table S4.** Frequency of clonal hematopoiesis mutations in the top 13 genes, stratified by radioactive iodine therapy status/dose and variant allele frequency range.
**Table S5.** Frequency of clonal hematopoiesis mutations in the top 13 genes based on only ones with maximum variant allele frequency (VAF) per individual, stratified by radioactive iodine therapy status/dose and VAF range.
**Table S6.** Overview of CH mutations of patients with paired samples before and after RAIT.

## Data Availability

The sequence data generated in this study are publicly available in the Sequence Read Archive (SRA) under the BioProject number PRJNA1078906 (https://dataview.ncbi.nlm.nih.gov/object/PRJNA1078906?reviewer=ra655d75492m6oqf49psr15j8i).

## References

[1] Haugen BR , Alexander EK , Bible KC , Doherty GM , Mandel SJ , Nikiforov YE , et al. 2015 American Thyroid Association management guidelines for adult patients with thyroid nodules and differentiated thyroid cancer: the American Thyroid Association guidelines task force on thyroid nodules and differentiated thyroid cancer. Thyroid. 2016;26:1–133. 10.1089/thy.2015.0020 26462967 PMC4739132

[2] Kim MJ , Cho SW , Kim YA , Choi HS , Park YJ , Park DJ , et al. Clinical outcomes of repeated radioactive iodine therapy for graves' disease. Endocrinol Metab. 2022;37:524–532. 10.3803/EnM.2022.1418 PMC9262691 35709827

[3] Tuttle RM , Ahuja S , Avram AM , Bernet VJ , Bourguet P , Daniels GH , et al. Controversies, consensus, and collaboration in the use of (131)I therapy in differentiated thyroid cancer: a joint statement from the American Thyroid Association, the European Association of Nuclear Medicine, the Society of Nuclear Medicine and Molecular Imaging, and the European thyroid association. Thyroid. 2019;29:461–470. 10.1089/thy.2018.0597 30900516

[4] Avram AM , Giovanella L , Greenspan B , Lawson SA , Luster M , Van Nostrand D , et al. SNMMI procedure standard/EANM practice guideline for nuclear medicine evaluation and therapy of differentiated thyroid cancer: abbreviated version. J Nucl Med. 2022;63:15N–35N. 35649660

[5] Pacini F , Fuhrer D , Elisei R , Handkiewicz‐Junak D , Leboulleux S , Luster M , et al. 2022 ETA consensus statement: what are the indications for post‐surgical radioiodine therapy in differentiated thyroid cancer? Eur Thyroid J. 2022;11:e210046. 10.1530/etj-21-0046 34981741 PMC9142814

[6] Sgouros G , Bodei L , McDevitt MR , Nedrow JR . Radiopharmaceutical therapy in cancer: clinical advances and challenges. Nat Rev Drug Discov. 2020;19:589–608. 10.1038/s41573-020-0073-9 32728208 PMC7390460

[7] Kitahara CM , Sosa JA . Understanding the ever‐changing incidence of thyroid cancer. Nat Rev Endocrinol. 2020;16:617–618. 10.1038/s41574-020-00414-9 32895503 PMC7476643

[8] Kim KJ , Kim KJ , Choi J , Kim NH , Kim SG . Linear association between radioactive iodine dose and second primary malignancy risk in thyroid cancer. J Natl Cancer Inst. 2023;115:695–702. 10.1093/jnci/djad040 36821433 PMC10248848

[9] Seo GH , Kong KA , Kim BS , Kang SY , Moon BS , Yoon HJ , et al. Radioactive iodine treatment for children and young adults with thyroid cancer in South Korea: a population‐based study. J Clin Endocrinol Metab. 2021;106:E2580–E2588. 10.1210/clinem/dgab192 33755732

[10] Hong CM , Son J , Hyun MK , Lee JW , Lee JT . Second primary malignancy after radioiodine therapy in thyroid cancer patient: a nationwide study. Nucl Med Mol Imaging 2010. 2023;57:1. 10.1007/s13139-023-00818-1 PMC10654320 37982105

[11] Iyer NG , Morris LGT , Tuttle RM , Shaha AR , Ganly I . Rising incidence of second cancers in patients with low‐risk (T1N0) thyroid cancer who receive radioactive iodine therapy. Cancer. 2011;117:4439–4446. 10.1002/cncr.26070 21432843 PMC3155861

[12] Pasqual E , Schonfeld S , Morton LM , Villoing D , Lee C , Gonzalez AB , et al. Association between radioactive iodine treatment for pediatric and young adulthood differentiated thyroid cancer and risk of second primary malignancies. J Clin Oncol. 2022;40:1439–1449. 10.1200/jco.21.01841 35044839 PMC9061144

[13] Kim M , Kim H , Park S , Joo J , Kim IJ , Kim BH . Risk factors for second primary malignancies following thyroid cancer: a nationwide cohort study. Eur J Endocrinol. 2022;186:561–571. 10.1530/Eje-21-1208 35286279

[14] Molenaar RJ , Sidana S , Radivoyevitch T , Advani AS , Gerds AT , Carraway HE , et al. Risk of hematologic malignancies after radioiodine treatment of well‐differentiated thyroid cancer. J Clin Oncol. 2018;36:1831–1839. 10.1200/Jco.2017.75.0232 29252123 PMC8462524

[15] Seo GH , Cho YY , Chung JH , Kim SW . Increased risk of leukemia after radioactive iodine therapy in patients with thyroid cancer: a nationwide, population‐based study in Korea. Thyroid. 2015;25:927–934. 10.1089/thy.2014.0557 26133388

[16] Matsuda Y , Uchimura A , Satoh Y , Kato N , Toshishige M , Kajimura J , et al. Spectra and characteristics of somatic mutations induced by ionizing radiation in hematopoietic stem cells. Proc Natl Acad Sci USA. 2023;120:e2216550120. 10.1073/pnas.2216550120 37018193 PMC10104525

[17] Jaiswal S , Natarajan P , Silver AJ , Gibson CJ , Bick AG , Shvartz E , et al. Clonal hematopoiesis and risk of atherosclerotic cardiovascular disease. N Engl J Med. 2017;377:111–121. 10.1056/NEJMoa1701719 28636844 PMC6717509

[18] Coombs CC , Zehir A , Devlin SM , Kishtagari A , Syed A , Jonsson P , et al. Therapy‐related clonal hematopoiesis in patients with non‐hematologic cancers is common and associated with adverse clinical outcomes. Cell Stem Cell. 2017;21:374–382. 10.1016/j.stem.2017.07.010 28803919 PMC5591073

[19] Bolton KL , Ptashkin RN , Gao T , Braunstein L , Devlin SM , Kelly D , et al. Cancer therapy shapes the fitness landscape of clonal hematopoiesis. Nat Genet. 2020;52:1219–1226. 10.1038/s41588-020-00710-0 33106634 PMC7891089

[20] Arends CM , Kopp K , Hablesreiter R , Estrada N , Christen F , Moll UM , et al. Dynamics of clonal hematopoiesis under DNA‐damaging treatment in patients with ovarian cancer. Leukemia. 2024;38:1378–1389. 10.1038/s41375-024-02253-3 38637689 PMC11147769

[21] Abelson S , Collord G , Ng SWK , Weissbrod O , Cohen NM , Niemeyer E , et al. Prediction of acute myeloid leukaemia risk in healthy individuals. Nature. 2018;559:400–404. 10.1038/s41586-018-0317-6 29988082 PMC6485381

[22] Desai P , Mencia‐Trinchant N , Savenkov O , Simon MS , Cheang G , Lee S , et al. Somatic mutations precede acute myeloid leukemia years before diagnosis. Nat Med. 2018;24:1015–1023. 10.1038/s41591-018-0081-z 29988143 PMC6849383

[23] Weeks LD , Niroula A , Neuberg D , Wong W , Lindsley RC , Luskin MR , et al. Prediction of risk for myeloid malignancy in clonal hematopoiesis. N Engl J Med Evid. 2023;2:EVIDoa2200310. 10.1056/EVIDoa2200310 PMC10361696 37483562

[24] Stonestrom AJ , Menghrajani KN , Devlin SM , Franch‐Expósito S , Ptashkin RN , Patel SY , et al. High‐risk and silent clonal hematopoietic genotypes in patients with nonhematologic cancer. Blood Adv. 2024;8:846–856. 10.1182/bloodadvances.2023011262 38147626 PMC10875331

[25] Hsu JI , Dayaram T , Tovy A , De Braekeleer E , Jeong MR , Wang F , et al. PPM1D mutations drive clonal hematopoiesis in response to cytotoxic chemotherapy. Cell Stem Cell. 2018;23:700–713. 10.1016/j.stem.2018.10.004 30388424 PMC6224657

[26] Boucai L , Falcone J , Ukena J , Coombs CC , Zehir A , Ptashkin R , et al. Radioactive iodine‐related clonal hematopoiesis in thyroid cancer is common and associated with decreased survival. J Clin Endocrinol Metab. 2018;103:4216–4223. 10.1210/jc.2018-00803 30137527 PMC6194804

[27] van Zeventer IA de Graaf AO Wouters HJCM van der van der Reijden BA Klauw MM de Witte T Jonker MA Malcovati L Jansen JH Huls G 2020 Mutational spectrum and dynamics of clonal hematopoiesis in anemia of older individuals Blood 135 1161 1170 10.1182/blood.2019004362 32243522

[28] Cochran JD , Yura Y , Thel MC , Doviak H , Polizio AH , Arai Y , et al. Clonal hematopoiesis in clinical and experimental heart failure with preserved ejection fraction. Circulation. 2023;148:1165–1178. 10.1161/Circulationaha.123.064170 37681311 PMC10575571

[29] Ortmann CA , Dorsheimer L , Abou‐El‐Ardat K , Hoffrichter J , Assmus B , Bonig H , et al. Functional dominance of CHIP‐mutated hematopoietic stem cells in patients undergoing autologous transplantation. Cell Rep. 2019;27:2022. 10.1016/j.celrep.2019.04.064 31091442

[30] Feusier JE , Arunachalam S , Tashi T , Baker MJ , VanSant‐Webb C , Ferdig A , et al. Large‐scale identification of clonal hematopoiesis and mutations recurrent in blood cancers. Blood Cancer Discov. 2021;2:226–237. 10.1158/2643-3230.Bcd-20-0094 34027416 PMC8133376

[31] Acuna‐Hidalgo R , Sengul H , Steehouwer M , van de Vorst M , Vermeulen SH , Kiemeney LALM , et al. Ultra‐sensitive sequencing identifies high prevalence of clonal hematopoiesis‐associated mutations throughout adult life. Am J Hum Genet. 2017;101:50–64. 10.1016/j.ajhg.2017.05.013 28669404 PMC5501773

[32] Bick AG , Weinstock JS , Nandakumar SK , Fulco CP , Bao EL , Zekavat SM , et al. Inherited causes of clonal haematopoiesis in 97,691 whole genomes. Nature. 2020;586:763–768. 10.1038/s41586-020-2819-2 33057201 PMC7944936

[33] Lai ZW , Markovets A , Ahdesmaki M , Chapman B , Hofmann O , McEwen R , et al. VarDict: a novel and versatile variant caller for next‐generation sequencing in cancer research. Nucleic Acids Res. 2016;44:e108. 10.1093/nar/gkw227 27060149 PMC4914105

[34] Papaemmanuil E , Gerstung M , Bullinger L , Gaidzik Verena I , Paschka P , Roberts Nicola D , et al. Genomic classification and prognosis in acute myeloid leukemia. N Engl J Med. 2016;374:2209–2221. 10.1056/NEJMoa1516192 27276561 PMC4979995

[35] Diossy M , Sztupinszki Z , Krzystanek M , Borcsok J , Eklund AC , Csabai I , et al. Strand orientation bias detector to determine the probability of FFPE sequencing artifacts. Brief Bioinform. 2021;22:bbab186. 10.1093/bib/bbab186 34015811

[36] Krusche P , Trigg L , Boutros PC , Mason CE , De La Vega FM , Moore BL , et al. Best practices for benchmarking germline small‐variant calls in human genomes. Nat Biotechnol. 2019;37:555–560. 10.1038/s41587-019-0054-x 30858580 PMC6699627

[37] Rentzsch P , Witten D , Cooper GM , Shendure J , Kircher M . CADD: predicting the deleteriousness of variants throughout the human genome. Nucleic Acids Res. 2019;47:D886–D894. 10.1093/nar/gky1016 30371827 PMC6323892

[38] Quang D , Chen YF , Xie XH . DANN: a deep learning approach for annotating the pathogenicity of genetic variants. Bioinformatics. 2015;31:761–763. 10.1093/bioinformatics/btu703 25338716 PMC4341060

[39] Shihab HA , Rogers MF , Gough J , Mort M , Cooper DN , Day INM , et al. An integrative approach to predicting the functional effects of non‐coding and coding sequence variation. Bioinformatics. 2015;31:1536–1543. 10.1093/bioinformatics/btv009 25583119 PMC4426838

[40] Sim NL , Kumar P , Hu J , Henikoff S , Schneider G , Ng PC . SIFT web server: predicting effects of amino acid substitutions on proteins. Nucleic Acids Res. 2012;40:W452–W457. 10.1093/nar/gks539 22689647 PMC3394338

[41] Reva B , Antipin Y , Sander C . Predicting the functional impact of protein mutations: application to cancer genomics. Nucleic Acids Res. 2011;39:E118–U185. 10.1093/nar/gkr407 21727090 PMC3177186

[42] Sundaram L , Gao H , Padigepati SR , McRae JF , Li YJ , Kosmicki JA , et al. Predicting the clinical impact of human mutation with deep neural networks. Nat Genet. 2018;50:1161–1170. 10.1038/s41588-018-0167-z 30038395 PMC6237276

[43] Dong CL , Wei P , Jian XQ , Gibbs R , Boerwinkle E , Wang K , et al. Comparison and integration of deleteriousness prediction methods for nonsynonymous SNVs in whole exome sequencing studies. Hum Mol Genet. 2015;24:2125–2137. 10.1093/hmg/ddu733 25552646 PMC4375422

[44] Liu XM , Wu CL , Li C , Boerwinkle E . dbNSFP v3.0: a one‐stop database of functional predictions and annotations for human nonsynonymous and splice‐site SNVs. Hum Mutat. 2016;37:235–241. 10.1002/humu.22932 26555599 PMC4752381

[45] Jagadeesh KA , Wenger AM , Berger MJ , Guturu H , Stenson PD , Cooper DN , et al. M‐CAP eliminates a majority of variants of uncertain significance in clinical exomes at high sensitivity. Nat Genet. 2016;48:1581–1586. 10.1038/ng.3703 27776117

[46] Ioannidis NM , Rothstein JH , Pejaver V , Middha S , McDonnell SK , Baheti S , et al. REVEL: An ensemble method for predicting the pathogenicity of rare missense variants. Am J Hum Genet. 2016;99:877–885. 10.1016/j.ajhg.2016.08.016 27666373 PMC5065685

[47] Martincorena I , Raine KM , Gerstung M , Dawson KJ , Haase K , Van Loo P , et al. Universal patterns of selection in cancer and somatic tissues. Cell. 2017;171:1029–1041. 10.1016/j.cell.2017.09.042 29056346 PMC5720395

[48] Kahn JD , Miller PG , Silver AJ , Sellar RS , Bhatt S , Gibson C , et al. PPM1D‐truncating mutations confer resistance to chemotherapy and sensitivity to PPM1D inhibition in hematopoietic cells. Blood. 2018;132:1095–1105. 10.1182/blood-2018-05-850339 29954749 PMC6137556

[49] Lindsley RC , Saber W , Mar BG , Redd R , Wang T , Haagenson MD , et al. Prognostic mutations in myelodysplastic syndrome after stem‐cell transplantation. N Engl J Med. 2017;376:536–547. 10.1056/NEJMoa1611604 28177873 PMC5438571

[50] Florez MA , Tran BT , Wathan TK , DeGregori J , Pietras EM , King KY . Clonal hematopoiesis: mutation‐specific adaptation to environmental change. Cell Stem Cell. 2022;29:882–904. 10.1016/j.stem.2022.05.006 35659875 PMC9202417

[51] Eskelund CW , Husby S , Favero F , Klausen TW , Rodriguez‐Gonzalez FG , Kolstad A , et al. Clonal hematopoiesis evolves from pretreatment clones and stabilizes after end of chemotherapy in patients with MCL. Blood. 2020;135:2000–2004. 10.1182/blood.2019003539 32181815

[52] Yun JK , Kim S , An H , Lee GD , Kim HR , Kim YH , et al. Pre‐operative clonal hematopoiesis is related to adverse outcome in lung cancer after adjuvant therapy. Genome Med. 2023;15:111. 10.1186/s13073-023-01266-4 38087308 PMC10714617

[53] Milsom MD . Potential pre‐leukemic mutations in PPM1D confer chemotherapy resistance to aged HSC clones. HemaSphere. 2019;3:e171. 10.1097/hs9.0000000000000171 31723810 PMC6745934

[54] Miller PG , Sperling AS , Mayerhofer C , McConkey ME , Ellegast JM , Da Silva C , et al. PPM1D modulates hematopoietic cell fitness and response to DNA damage and is a therapeutic target in myeloid malignancy. Blood. 2023;142:2079–2091. 10.1182/blood.2023020331 37595362 PMC10733824

[55] Burocziova M , Danek P , Oravetzova A , Chalupova Z , Alberich‐Jorda M , Macurek L . Ppm1d truncating mutations promote the development of genotoxic stress‐induced AML. Leukemia. 2023;37:2209–2220. 10.1038/s41375-023-02030-8 37709843 PMC10624630

[56] Gibson CJ , Lindsley RC , Tchekmedyian V , Mar BG , Shi J , Jaiswal S , et al. Clonal hematopoiesis associated with adverse outcomes after autologous stem‐cell transplantation for lymphoma. J Clin Oncol. 2017;35:1598–1605. 10.1200/jco.2016.71.6712 28068180 PMC5455707

[57] Fandrei D , Pegliasco J , Pasquier F , Ibrahim N , Kfoury M , Berthon C , et al. Clonal evolution of PPM1D mutations in the Spectrum of myeloid disorders. Clin Cancer Res. 2025;31:2241–2253. 10.1158/1078-0432.Ccr-24-3683 40162927

[58] Yura Y , Miura‐Yura E , Katanasaka Y , Min K‐D , Chavkin N , Polizio AH , et al. The cancer therapy‐related clonal hematopoiesis driver gene Ppm1d promotes inflammation and non‐ischemic heart failure in mice. Circ Res. 2021;129:684–698. 10.1161/CIRCRESAHA.121.319314 34315245 PMC8409899

[59] Akamandisa MP , Nie K , Nahta R , Hambardzumyan D , Castellino RC . Inhibition of mutant PPM1D enhances DNA damage response and growth suppressive effects of ionizing radiation in diffuse intrinsic pontine glioma. Neuro Oncol. 2019;21:786–799. 10.1093/neuonc/noz053 30852603 PMC6556861

[60] Boucai L , Ptashkin RN , Levine RL , Fagin JA . Effects of radioactive iodine on clonal hematopoiesis in patients with thyroid cancer: a prospective study. Clin Endocrinol. 2023;99:122–129. 10.1111/cen.14925 PMC10644358 37088956

[61] de Haan G , Lazare SS . Aging of hematopoietic stem cells. Blood. 2018;131:479–487. 10.1182/blood-2017-06-746412 29141947

[62] Silver AJ , Bick AG , Savona MR . Germline risk of clonal haematopoiesis. Nat Rev Genet. 2021;22:603–617. 10.1038/s41576-021-00356-6 33986496 PMC8117131

[63] Tiedje V , Vela PS , Yang JL , Untch BR , Boucai L , Stonestrom AJ , et al. Targetable treatment resistance in thyroid cancer with clonal hematopoiesis. *bioRxiv* . 2024 10.1101/2024.10.10.617685

[64] Young AL , Challen GA , Birmann BM , Druley TE . Clonal haematopoiesis harbouring AML‐associated mutations is ubiquitous in healthy adults. Nat Commun. 2016;7:12484. 10.1038/ncomms12484 27546487 PMC4996934

