# Supplementary material for: Association of high‐dose radioactive iodine therapy with PPM1D‐mutated clonal hematopoiesis in older individuals
Source: Mol Oncol. 2025 Jun 26;19(11):3079–95. doi: 10.1002/1878-0261.70078 (PMC12591306; doi:10.1002/1878-0261.70078)
Supplement: Supplementary file 1 — Fig. S1. Distribution of radioactive iodine therapy dose in our study cohort. Fig. S2. Determination of radioactive iodine therapy dose cut‐off for low and high groups. Fig. S3. Distribution of coverage depth for detected clonal hematopoiesis mutations. Fig. S4. Distribution of the variant allele frequency of detected all clonal hematopoiesis mutations. Fig. S5. Association of age and radioactive iodine therapy status/dose with clonal hematopoiesis. Fig. S6. Prevalence of putative driver mutations associated with age‐related clonal hematopoiesis (ARCH‐PD) at various variant allele frequency thresholds. Fig. S7. The number of clonal hematopoiesis mutations per individual across radioactive iodine therapy status/dose groups at various variant allele frequency cutoffs. Fig. S8. Association of age and radioactive iodine therapy dose with variant allele frequency of clonal hematopoiesis. Fig. S9. Multivariable linear regression analyses for maximum variant allele frequency. Fig. S10. Multivariable logistic analyses examining the presence clonal hematopoiesis with various variant allele frequency cut‐offs. Fig. S11. Oncoplots illustrating CH mutations in the top 13 genes at various variant allele frequency cut‐offs. Fig. S12. Lollipop plots highlighting mutation positions and frequencies in TET2, DNMT3A, TP53 genes. Fig. S13. Multivariable logistic regression analysis for the association of clonal hematopoiesis mutations in each gene (excluding the top 13 genes) with clinical variables. Fig. S14. Multivariable logistic analyses for the presence of DNA damage response genes (PPM1D and TP53) mutated clonal hematopoiesis with various variant allele frequency cut‐offs. Fig. S15. Multivariable logistic analyses for the presence of DTA genes (DNMT3A, TET2, and ASXL1) mutated clonal hematopoiesis with various variant allele frequency cut‐offs. Fig. S16. Relationship between variant allele frequency of clonal hematopoiesis mutations and predicted pathogenicity scores. Fig. S17. A [file MOL2-19-3079-s001.docx]

**Supplemental Materials**

**Association of high-dose radioactive iodine therapy with PPM1D-mutated clonal hematopoiesis in older individuals**

**Supplementary figures**

**
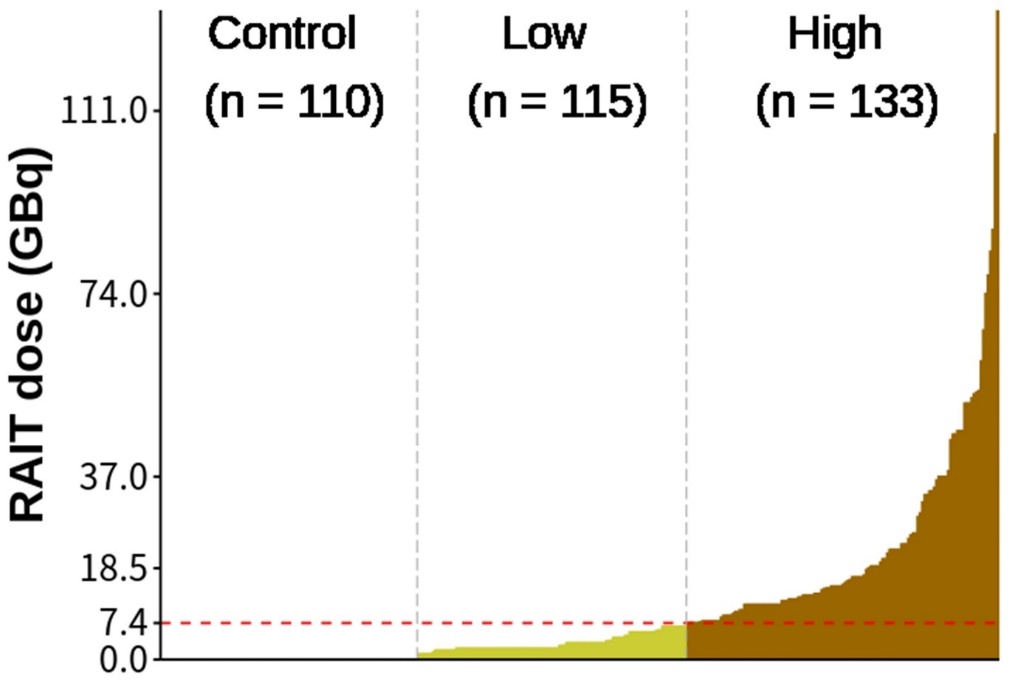
**

**Fig. S1**. **Distribution of radioactive iodine therapy dose in our study cohort.** The patients were categorized into three distinct groups based on their radioactive iodine therapy (RAIT) status or dose: control (patients who did not receive RAIT), low (patients who received a RAIT dose below 7.4 GBq), and high (patients who received a RAIT dose equal to or above 7.4 GBq).

**Fig. S2. Determination of radioactive iodine therapy dose cut-off for low and high groups.** To facilitate the assessment of dose-dependent effects of received radioactive iodine therapy (RAIT), we categorized patients based on RAIT dose. **(A)** For patients who received RAIT, the median dose was 7.77 GBq (red dashed line). **(B)** Area under the curve (AUC) analysis was conducted to predict clonal hematopoiesis, defined by a variant allele frequency (VAF) exceeding 2% or 5%, with resulting cut-offs established at 9.9 GBq for both VAF thresholds.

**Fig. S3**. **Distribution of coverage depth for detected clonal hematopoiesis mutations.** Upon deduplication using unique molecular identifiers, the median sequencing depth for identified clonal hematopoiesis (CH) mutations reached 1926x, ensuring highly sensitive detection of mutations with low variant allele frequencies.

**
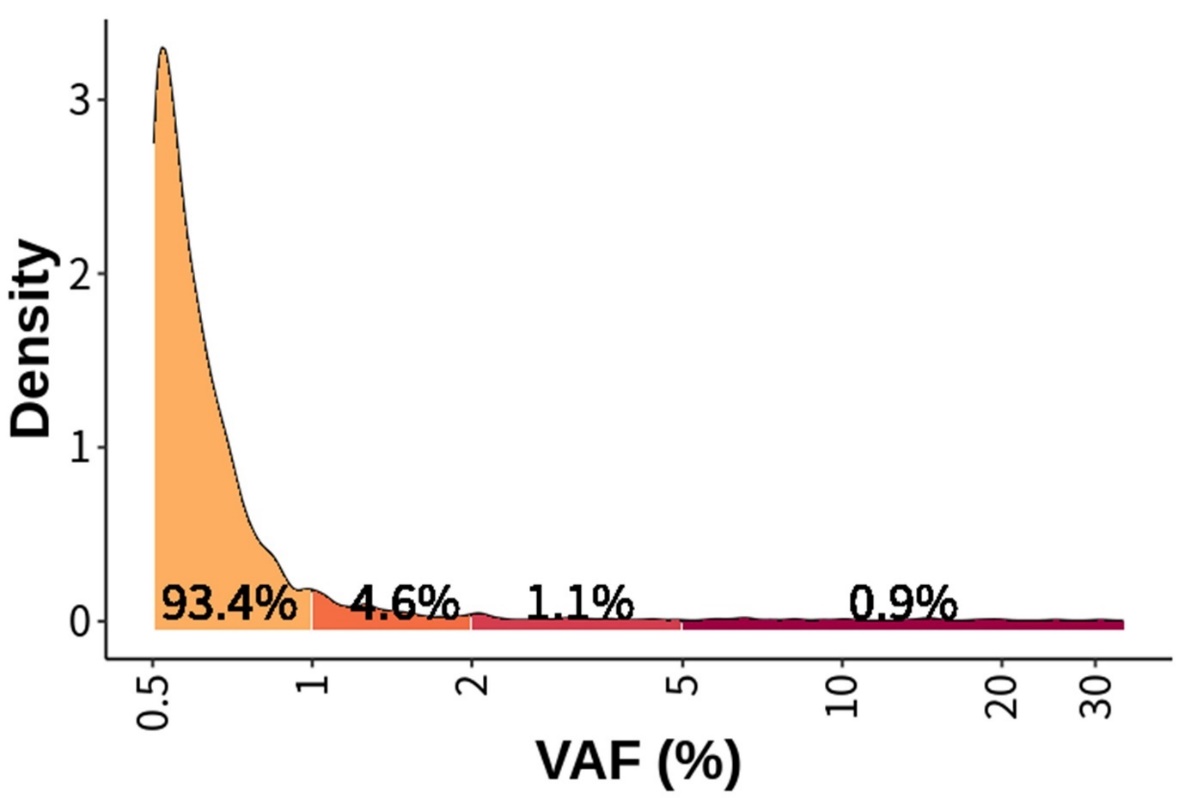
**

**Fig. S4**. **Distribution of the variant allele frequency of detected all clonal hematopoiesis mutations**. It displayed a right-skewed pattern, with the majority of mutations having a variant allele frequency (VAF) less than 1%.

**Fig. S5**. **Association of age and radioactive iodine therapy status/dose with clonal hematopoiesis.** **(A)** The prevalence of clonal hematopoiesis (CH), defined by a variant allele frequency (VAF) exceeding 2%, was generally higher in older patients across all RAIT status/dose groups when investigated by age at CH test (Age_CH_) subgroups by decades. **(B)** Mutations with VAF levels were more prevalent in older individuals, particularly within the high RAIT dose group when investigated by age_CH_ subgroups by decades.


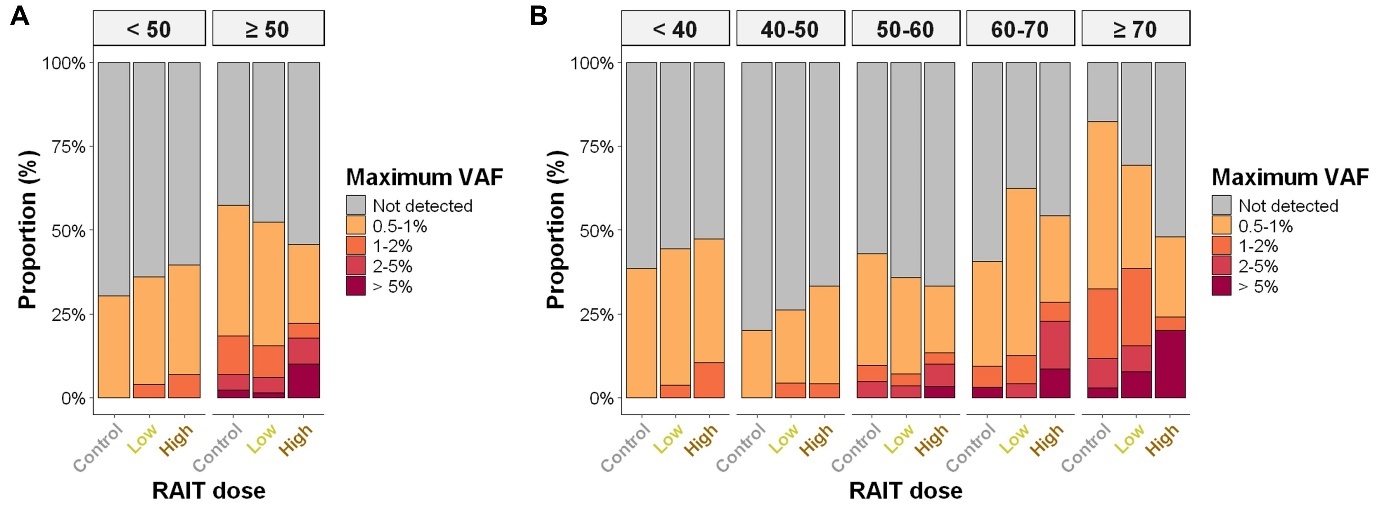


**Fig. S6. Prevalence of putative driver mutations associated with age-related clonal hematopoiesis (ARCH-PD) at various variant allele frequency thresholds. (A)** Stratified by age at CH test (using a 50-year cutoff) and **(B)** stratified by decades in each RAIT status/dose group.

**Fig. S7.** **The number of clonal hematopoiesis mutations per individual across radioactive iodine therapy status/dose groups at various variant allele frequency cutoffs.** Only at variant allele frequency (VAF) cutoff of 5%, a statistically significant difference was observed in the high-dose radioactive iodine therapy (RAIT) group for older individuals (Age at CH test; Age_CH_ ≥ 50 years). The pairwise Wilcoxon test was used for statistical comparisons.

** Fig. S8**. **Association of age and radioactive iodine therapy dose with variant allele frequency of clonal hematopoiesis.** **(A)** Linear regression analysis demonstrated a significant linear relationship between age at clonal hematopoiesis test (Age_CH_) and maximum variant allele frequency (VAF). The colored horizontal dashed lines represent the mean of the maximum VAF for each radioactive iodine therapy (RAIT) status/dose group, corresponding to their respective colors. **(B)** Linear regression analysis demonstrated a significant linear relationship between RAIT dose and maximum VAF. The VAF for non-detected samples was estimated to be 0.25%.


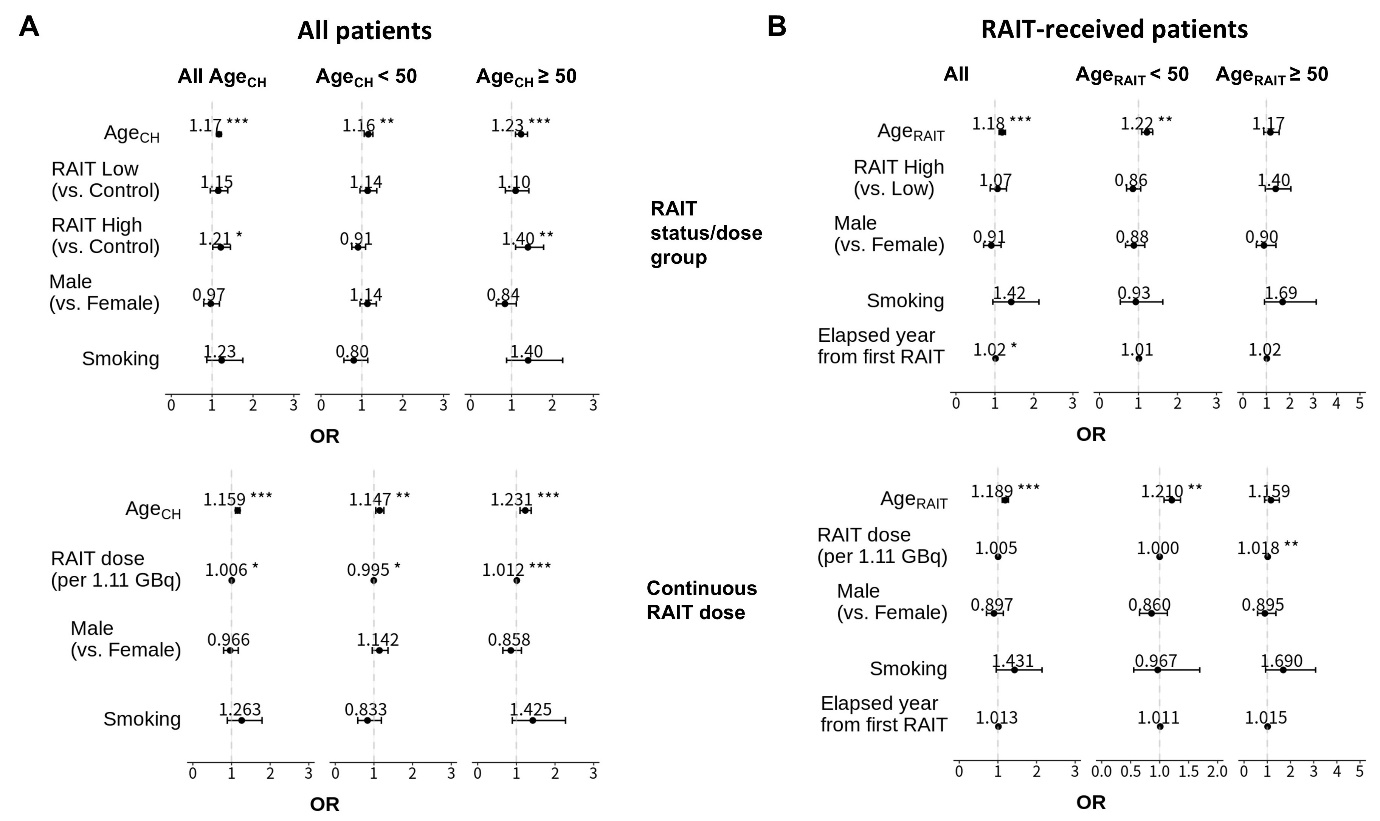


**Fig. S9**. **Multivariable linear regression analyses for maximum variant allele frequency.  (A)** Multivariable linear regression analyses for the maximum variant allele frequency (VAF) of clonal hematopoiesis (CH). Independent variables included age at CH test (Age_CH_), sex, smoking, and radioactive iodine therapy (RAIT) status/dose group (top panel), or continuous RAIT dose (bottom panel), with subgrouping by Age_CH_. **(B)** Multivariable linear regression analyses for the maximum VAF of CH in association with age at first RAIT (Age_RAIT_), sex, smoking, and radioactive iodine therapy (RAIT) status/dose group (top panel), or continuous RAIT dose (bottom panel), with subgrouping by Age_RAIT_. Only individuals who received RAIT were included in these analyses. The maximum VAF is defined as the VAF of the mutation with the highest frequency in an individual. In regression analyses, VAF was log-transformed. The unit of age and RAIT dose is per 10 years and per 1.11 GBq increase, respectively. Error bars indicate confidence interval. *: p-value < 0.05; **: p-value < 0.01; ***: p-value < 0.001.

**Fig. S10**. **Multivariable logistic analyses examining the presence clonal hematopoiesis with various variant allele frequency cut-offs.** Independent variables included age at CH test (Age_CH_), radioactive iodine therapy (RAIT) status/dose group, sex, and smoking, with subgrouping by Age_CH_. Firth logistic regression was used because of the sparse data. The maximum VAF is defined as the VAF of the mutation with the highest frequency in an individual. The unit of Age_CH_ is per 10 years increase. Error bars indicate confidence interval. *: p-value < 0.05; **: p-value < 0.01; ***: p-value < 0.001.

**Fig. S11**. **Oncoplots illustrating CH mutations in the top 13 genes at various variant allele frequency cut-offs.** Oncoplots were generated to visually represent the distribution of clonal hematopoiesis (CH) mutations in the top 13 genes, with maximum variant allele frequency (VAF) cut-offs set at 0.5%, 1%, 2%, and 5%. These plots illustrate mutation types within each radioactive iodine therapy (RAIT) status/dose group, while considering mutation count per individual, age at CH test, RAIT dose, and sex. The maximum VAF is defined as the VAF of the mutation with the highest frequency in an individual.

**Fig. S12. Lollipop plots highlighting mutation positions and frequencies in *TET2, DNMT3A, TP53* genes**. The variant allele frequency of corresponding positions and their frequency densities (gray background) are represented.

**Fig. S13. Multivariable logistic regression analysis for the association of clonal hematopoiesis mutations in each gene (excluding the top 13 genes) with clinical variables.** Analyses were conducted using mutation presence (VAF > 0.5%) in each gene as the dependent variable. Independent variables included age at CH test (Age_CH_; adjusted for sex, smoking, prior RAIT), age at first RAIT (Age_RAIT_; adjusted for sex, smoking, RAIT dose, elapsed time from first RAIT), prior RAIT (adjusted for Age_CH_, sex, smoking), and RAIT dose (adjusted for Age_CH_, sex, smoking, elapsed time from first RAIT). Non-significant results (p-value over 0.05) are presented as gray dots. Error bars indicate confidence interval.


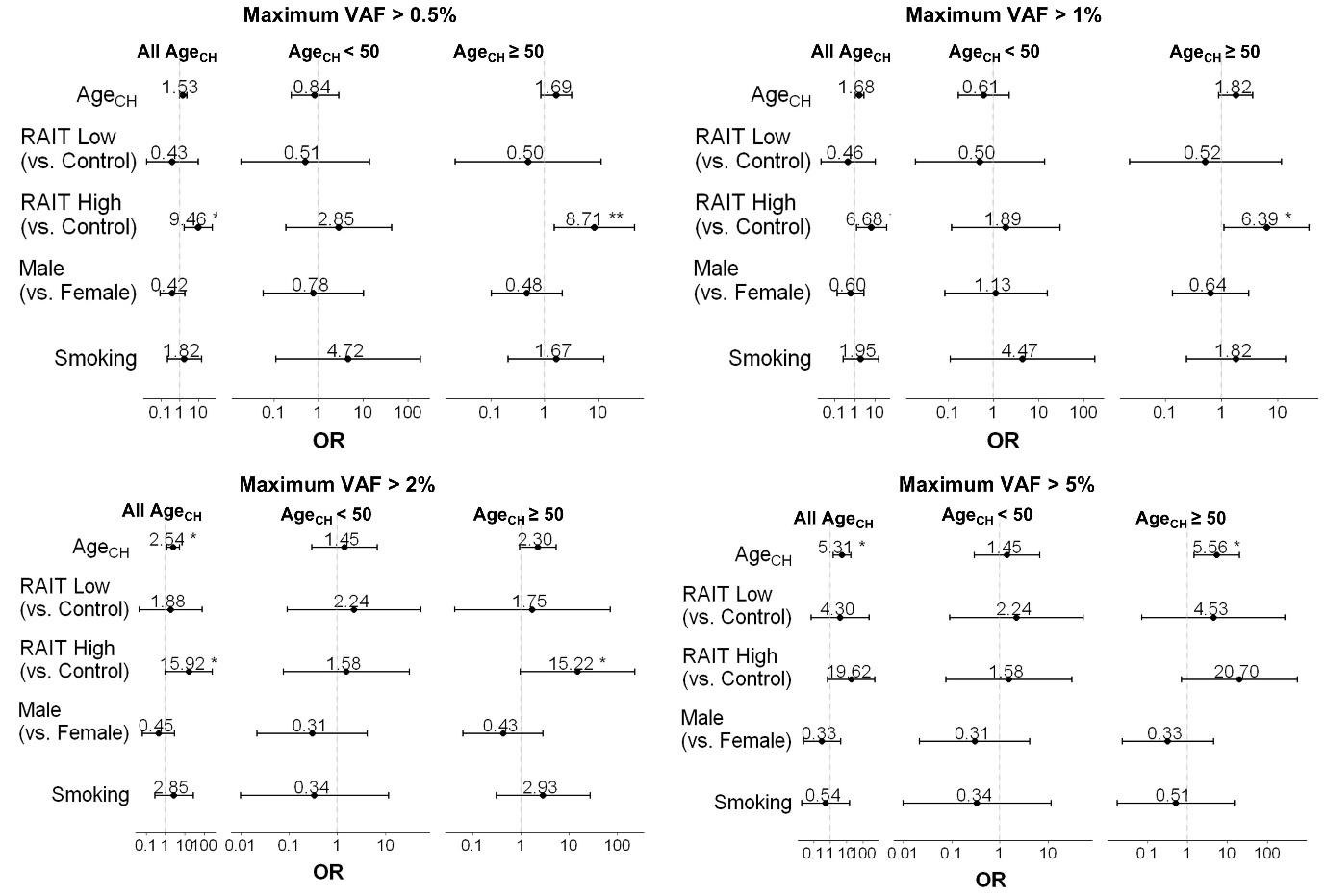
**Fig. S14**. **Multivariable logistic analyses for the presence of DNA damage response genes (*PPM1D* and *TP53*) mutated clonal hematopoiesis with various variant allele frequency cut-offs.** Independent variables included age at CH test (Age_CH_), radioactive iodine therapy (RAIT) status/dose group, sex, and smoking, with subgrouping by Age_CH_. Firth logistic regression was used because of the sparse data. The maximum VAF is defined as the VAF of the mutation with the highest frequency in an individual. The unit of Age_CH_ is per 10 years increase. Error bars indicate confidence interval. *: p-value < 0.05; **: p-value < 0.01; ***: p-value < 0.001.


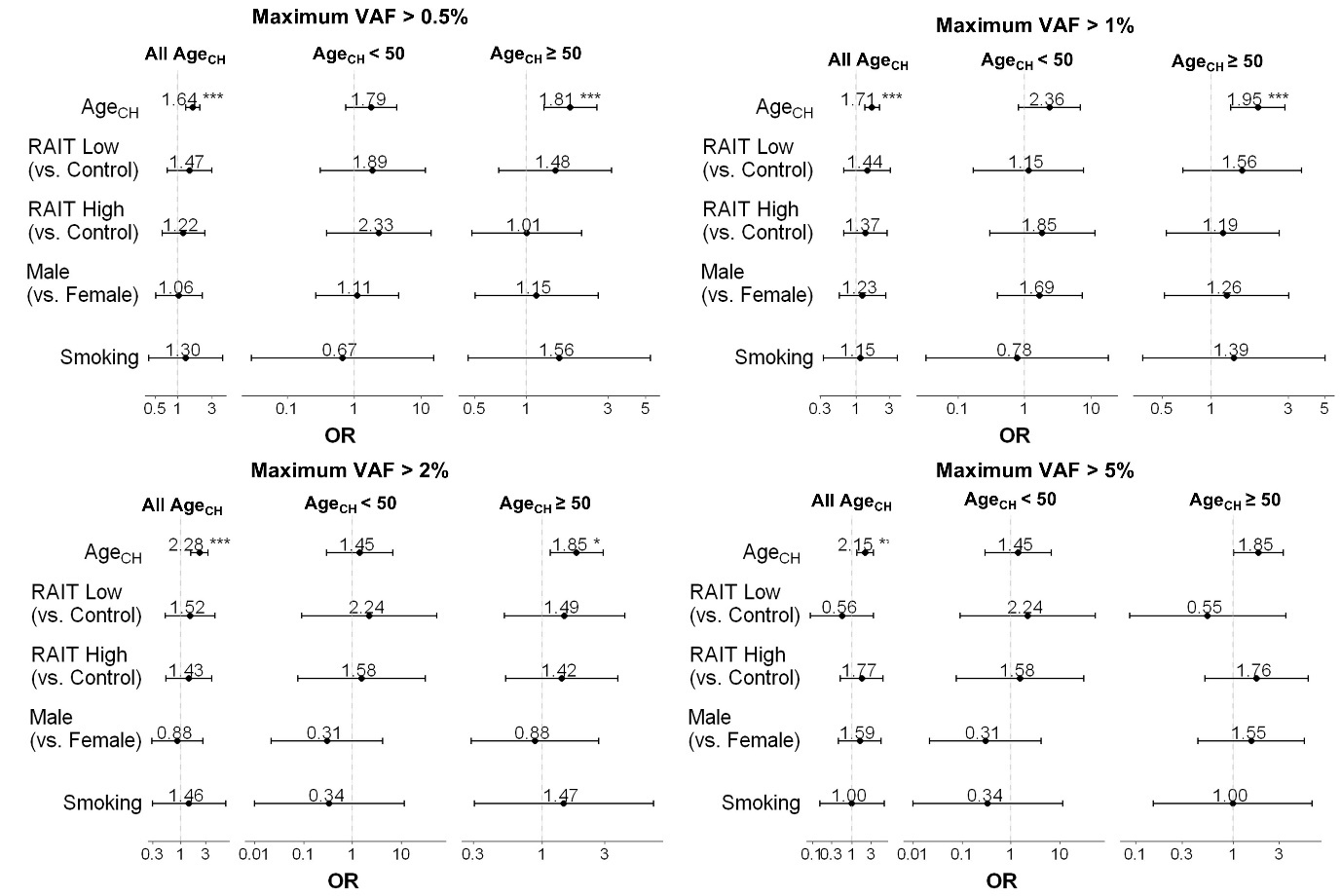


**Fig. S15**. **Multivariable logistic analyses for the presence of DTA genes (*DNMT3A, TET2, and ASXL1*) mutated clonal hematopoiesis with various variant allele frequency cut-offs.** Independent variables included age at CH test (Age_CH_), radioactive iodine therapy (RAIT) status/dose group, sex, and smoking, with subgrouping by Age_CH_. Firth logistic regression was used because of the sparse data. The maximum VAF is defined as the VAF of the mutation with the highest frequency in an individual. The unit of Age_CH_ is per 10 years increase. Error bars indicate confidence interval. *: p-value < 0.05; **: p-value < 0.01; ***: p-value < 0.001.

**Fig. S16.** **Relationship between variant allele frequency of clonal hematopoiesis mutations and predicted pathogenicity scores.** We investigated the correlation between the variant allele frequency (VAF) of clonal hematopoiesis (CH) and predicted pathogenicity scores determined by various in-silico tools, including **(A)** CADD, **(B)** DANN, **(C)** fathmm-MKL, **(D)** SIFT, **(E)** MutationAssessor, **(F)** PrimateAI, **(G)** PROVEAN, **(H)** MetaLR, **(I)** MetaSVM, **(J)** M-CAP, and **(K)** REVEL. In general, a positive correlation is observed across these tools.

**Fig. S17.** **Analysis of nonsynonymous to synonymous substitution ratio** **(dN/dS) in clonal hematopoiesis-related genes across age groups.** Notably, positive selection in specific clonal hematopoiesis (CH)-related genes is observed exclusively in older individuals (age at CH test; age_CH_ ≥ 50 years).

**Fig. S18.** **Mutual exclusivity and co-occurrence of genes in CH with various VAF cut-offs.** We generated heatmaps illustrating patterns of mutual exclusivity or co-occurrence of mutations in genes associated with clonal hematopoiesis (CH). The analysis is conducted using variant allele frequency (VAF) cut-offs of 0.5% (top 25 genes), 1%, 2%, and 5%. The maximum VAF is defined as the VAF of the mutation with the highest frequency in an individual. The numbers in brackets represent the count of mutations for the corresponding genes.

**Supplementary tables**

**Table S1.** A list of clonal hematopoiesis-related genes targeted in this study

| *ABL1* | *CSF3R* | *IDH2* | *PHF6* | *STAG2* |
| --- | --- | --- | --- | --- |
| *AKT1* | *CUX1* | *IKZF1* | *PIK3CA* | *STAT3* |
| *ASXL1* | *DDX41* | *JAK1* | *PIK3CD* | *STAT5B* |
| *ASXL2* | *DHX15* | *JAK2* | *PIK3R1* | *TERT* |
| *ATM* | *DNM2* | *JAK3* | *PPM1D* | *TET2* |
| *ATRX* | *DNMT3A* | *KDM6A* | *PPP4C* | *TP53* |
| *BCOR* | *EP300* | *KIT* | *PTEN* | *TRIM24* |
| *BCORL1* | *ETV6* | *KMT2A* | *PTPN11* | *TRRAP* |
| *BRAF* | *EZH2* | *KRAS* | *RAD21* | *U2AF1* |
| *BRCC3* | *FBXW7* | *MED12* | *RIT1* | *USP7* |
| *CALR* | *FLT3* | *MPL* | *RUNX1* | *WHSC1* |
| *CBL* | *GATA1* | *MYC* | *SETBP1* | *WT1* |
| *CBLB* | *GATA2* | *MYD88* | *SETD2* | *ZRSR2* |
| *CBLC* | *GATA3* | *NF1* | *SF3B1* |  |
| *CCND2* | *GIGYF2* | *NOTCH1* | *SH2B3* |  |
| *CDKN2A* | *GNAS* | *NPM1* | *SMARCA2* |  |
| *CEBPA* | *GNB1* | *NRAS* | *SMC1A* |  |
| *CHD4* | *HDAC7* | *NSD1* | *SMC3* |  |
| *CNOT3* | *HRAS* | *PAX5* | *SPI1* |  |
| *CREBBP* | *IDH1* | *PDGFRA* | *SRSF2* |  |

**Table S2.** Patient characteristics harboring clonal hematopoiesis mutations with variant allele frequency exceeding 5%.

| **No.** | **Sex** | **Age_CH_ (years)** | **Age_RAIT_ (years)** | **RAIT dose group** | **RAIT dose (GBq)** | **Smoking** | **Histology** | **WBC (10^9^/L)** | **Gene** | **Protein change** | **VAF (%)** |
| --- | --- | --- | --- | --- | --- | --- | --- | --- | --- | --- | --- |
| **#1** | F | 51 | NA | Control | NA | No | PTC | NA | *TET2* | p.C1875R | 6.6 |
| **#2** | M | 69 | NA | Control | NA | No | PTC | 3.82 | *TET2* | p.H629fs | 6.7 |
| **#3** | F | 84 | NA | Control | NA | No | Others | 7.44 | *DNMT3A* | p.Q656P | 9.2 |
| **#4** | F | 91 | NA | Control | NA | No | PTC | 3.45 | *TET2* | p.F1429fs | 14.3 |
|  |  |  |  |  |  |  |  |  | *TET2* | p.Q705fs | 14.7 |
| **#5** | F | 77 | 45 | Low | 2.78 | No | PTC | NA | *TET2* | p.E1428fs | 8.1 |
| **#6** | F | 51 | 46 | High | 51.8 | No | PTC | 12.08 | *TET2* | p.V1157M | 15.1 |
| **#7** | F | 56 | 44 | High | 9.99 | No | PTC | 5.64 | *CBL* | p.G289V | 25.3 |
| **#8** | M | 60 | 58 | High | 7.4 | Yes | PTC | NA | *RAD21* | p.Q613* | 13.3 |
| **#9** | F | 63 | 51 | High | 11.8 | No | PTC | 6.81 | *DNMT3A* | p.D712fs | 5.6 |
| **#10** | F | 65 | 63 | High | 12.2 | No | PTC | 4.98 | *ASXL1* | p.Q829* | 7.3 |
| **#11** | F | 65 | 43 | High | 87 | No | PTC | 8.76 | *TET2* | p.S56fs | 10 |
| **#12** | M | 75 | 54 | High | 34 | Yes | FTC | 6.39 | *DNMT3A* | p.T834fs | 14.3 |
| **#13** | F | 69 | 65 | High | 11.1 | No | Others | 11.12 | *RAD21* | p.D536del | 10.2 |
| **#14** | M | 70 | 59 | High | 54.4 | No | PTC | NA | *PPM1D* | p.W427* | 6.4 |
|  |  |  |  |  |  |  |  |  | *PPM1D* | p.L484fs | 18.1 |
|  |  |  |  |  |  |  |  |  | *DNMT3A* | p.K841fs | 19.9 |
|  |  |  |  |  |  |  |  |  | *TET2* | p.F1377I | 30.7 |
| **#15** | M | 71 | 64 | High | 46.3 | No | FTC | 6.09 | *DNMT3A* | p.N802fs | 6.1 |
| **#16** | F | 76 | 59 | High | 20.4 | No | FTC | NA | *PPM1D* | p.L484fs | 8.2 |
| **#17** | F | 77 | 65 | High | 54 | No | FTC | 8.98 | *TP53* | p.S240C | 5.8 |
|  |  |  |  |  |  |  |  |  | *PPM1D* | p.I526fs | 6.6 |
|  |  |  |  |  |  |  |  |  | *PPM1D* | p.R552* | 19.1 |

Abbreviations: RAIT (radioactive iodine therapy); Age_CH_ (age at clonal hematopoiesis test); Age_RAIT_ (age at the first session of RAIT); VAF (variant allele frequency)

**Table S3.** Interaction terms between age at clonal hematopoiesis test (AgeCH) and radioactive iodine therapy dose or status/dose group

|  | OR | Lower 95% CI | Upper 95% CI | p-value | |
| --- | --- | --- | --- | --- | --- |
| **Age_CH_: RAIT dose (per 1.11 GBq)** | 1.0003 | 1.0001 | 1.0004 | | 5.05E-04 |
| **Age_CH_: Low-dose RAIT (vs. Control)** | 0.9987 | 0.9932 | 1.0042 | | 0.64 |
| **Age_CH_: High-dose RAIT group (vs. Control)** | 1.0041 | 0.9988 | 1.0095 | | 0.13 |

Abbreviations: Age_CH_ (age at clonal hematopoiesis test)**;** RAIT (radioactive iodine therapy)

**Table S4.** Frequency of clonal hematopoiesis mutations in the top 13 genes, stratified by radioactive iodine therapy status/dose and variant allele frequency range.

| **Gene** | **RAIT status/dose** | **VAF range** | | | | | **Total** |
| --- | --- | --- | --- | --- | --- | --- | --- |
|  |  | **< 0.5%** | **0.5-1%** | **1-2%** | **2-5%** | **> 5%** |  |
| *TET2* | Control | 0 (0.0) | 26 (46.4) | 5 (23.8) | 4 (50.0) | 4 (50.0) | 39 (40.6) |
|  | Low | 2 (66.7) | 17 (30.4) | 9 (42.9) | 2 (25.0) | 1 (12.5) | 31 (32.3) |
|  | High | 1 (33.3) | 13 (23.2) | 7 (33.3) | 2 (25.0) | 3 (37.5) | 26 (27.1) |
| *DNMT3A* | Control | 1 (50.0) | 35 (33.7) | 7 (31.8) | 0 (0.0) | 1 (20.0) | 44 (31.7) |
|  | Low | 1 (50.0) | 35 (33.7) | 7 (31.8) | 4 (66.7) | 0 (0.0) | 47 (33.8) |
|  | High | 0 (0.0) | 34 (32.7) | 8 (36.4) | 2 (33.3) | 4 (80.0) | 48 (34.5) |
| *PPM1D* | Control | 0 (0.0) | 2 (11.8) | 0 (0.0) | 0 (0.0) | 0 (0.0) | 2 (6.7) |
|  | Low | 1 (100.0) | 6 (35.3) | 0 (0.0) | 0 (0.0) | 0 (0.0) | 7 (23.3) |
|  | High | 0 (0.0) | 9 (52.9) | 5 (100.0) | 2 (100.0) | 5 (100.0) | 21 (70.0) |
| *TP53* | Control | 0 (NA) | 1 (7.1) | 1 (33.3) | 0 (0.0) | 0 (0.0) | 2 (9.1) |
|  | Low | 0 (NA) | 4 (28.6) | 0 (0.0) | 0 (0.0) | 0 (0.0) | 4 (18.2) |
|  | High | 0 (NA) | 9 (64.3) | 2 (66.7) | 4 (100.0) | 1 (100.0) | 16 (72.7) |
| *ASXL1* | Control | 1 (33.3) | 4 (17.4) | 0 (0.0) | 0 (0.0) | 0 (0.0) | 5 (16.7) |
|  | Low | 0 (0.0) | 13 (56.5) | 0 (0.0) | 0 (0.0) | 0 (0.0) | 13 (43.3) |
|  | High | 2 (66.7) | 6 (26.1) | 2 (100.0) | 1 (100.0) | 1 (100.0) | 12 (40.0) |
| *CBL* | Control | 0 (NA) | 2 (20.0) | 0 (NA) | 1 (100.0) | 0 (0.0) | 3 (25.0) |
|  | Low | 0 (NA) | 4 (40.0) | 0 (NA) | 0 (0.0) | 0 (0.0) | 4 (33.3) |
|  | High | 0 (NA) | 4 (40.0) | 0 (NA) | 0 (0.0) | 1 (100.0) | 5 (41.7) |
| *RAD21* | Control | 0 (0.0) | 2 (25.0) | 0 (NA) | 0 (NA) | 0 (0.0) | 2 (18.2) |
|  | Low | 1 (100.0) | 3 (37.5) | 0 (NA) | 0 (NA) | 0 (0.0) | 4 (36.4) |
|  | High | 0 (0.0) | 3 (37.5) | 0 (NA) | 0 (NA) | 2 (100.0) | 5 (45.5) |
| *ATM* | Control | 0 (0.0) | 17 (45.9) | 1 (50.0) | 1 (100.0) | 0 (NA) | 19 (46.3) |
|  | Low | 0 (0.0) | 11 (29.7) | 0 (0.0) | 0 (0.0) | 0 (NA) | 11 (26.8) |
|  | High | 1 (100.0) | 9 (24.3) | 1 (50.0) | 0 (0.0) | 0 (NA) | 11 (26.8) |
| *FBXW7* | Control | 1 (100.0) | 6 (50.0) | 0 (NA) | 0 (0.0) | 0 (NA) | 7 (50.0) |
|  | Low | 0 (0.0) | 3 (25.0) | 0 (NA) | 0 (0.0) | 0 (NA) | 3 (21.4) |
|  | High | 0 (0.0) | 3 (25.0) | 0 (NA) | 1 (100.0) | 0 (NA) | 4 (28.6) |
| *GNAS* | Control | 0 (0.0) | 21 (46.7) | 0 (0.0) | 0 (0.0) | 0 (NA) | 21 (43.8) |
|  | Low | 0 (0.0) | 13 (28.9) | 0 (0.0) | 1 (100.0) | 0 (NA) | 14 (29.2) |
|  | High | 1 (100.0) | 11 (24.4) | 1 (100.0) | 0 (0.0) | 0 (NA) | 13 (27.1) |
| *GNB1* | Control | 0 (NA) | 1 (20.0) | 0 (NA) | 0 (0.0) | 0 (NA) | 1 (16.7) |
|  | Low | 0 (NA) | 3 (60.0) | 0 (NA) | 1 (100.0) | 0 (NA) | 4 (66.7) |
|  | High | 0 (NA) | 1 (20.0) | 0 (NA) | 0 (0.0) | 0 (NA) | 1 (16.7) |
| *RUNX1* | Control | 0 (NA) | 13 (28.3) | 5 (50.0) | 1 (100.0) | 0 (NA) | 19 (33.3) |
|  | Low | 0 (NA) | 21 (45.7) | 4 (40.0) | 0 (0.0) | 0 (NA) | 25 (43.9) |
|  | High | 0 (NA) | 12 (26.1) | 1 (10.0) | 0 (0.0) | 0 (NA) | 13 (22.8) |
| *SH2B3* | Control | 0 (0.0) | 11 (44.0) | 1 (33.3) | 0 (0.0) | 0 (NA) | 12 (40.0) |
|  | Low | 0 (0.0) | 7 (28.0) | 1 (33.3) | 1 (100.0) | 0 (NA) | 9 (30.0) |
|  | High | 1 (100.0) | 7 (28.0) | 1 (33.3) | 0 (0.0) | 0 (NA) | 9 (30.0) |

Values are presented as count (percentage); “NA” indicates not applicable.

Abbreviations: RAIT (radioactive iodine therapy); VAF (variant allele frequency)

**Table S5.** Frequency of clonal hematopoiesis mutations in the top 13 genes based on only ones with maximum variant allele frequency (VAF) per individual, stratified by radioactive iodine therapy status/dose and VAF range.

| **Gene** | **RAIT status/dose** | **VAF range** | | | | | **Total** |
| --- | --- | --- | --- | --- | --- | --- | --- |
|  |  | **< 0.5%** | **0.5-1%** | **1-2%** | **2-5%** | **> 5%** |  |
| *TET2* | Control | 0 (NA) | 3 (60.0) | 3 (23.1) | 4 (57.1) | 3 (42.9) | 13 (40.6) |
|  | Low | 0 (NA) | 1 (20.0) | 5 (38.5) | 2 (28.6) | 1 (14.3) | 9 (28.1) |
|  | High | 0 (NA) | 1 (20.0) | 5 (38.5) | 1 (14.3) | 3 (42.9) | 10 (31.2) |
| *DNMT3A* | Control | 0 (NA) | 1 (12.5) | 5 (31.2) | 0 (0.0) | 1 (25.0) | 7 (21.2) |
|  | Low | 0 (NA) | 4 (50.0) | 4 (25.0) | 4 (80.0) | 0 (0.0) | 12 (36.4) |
|  | High | 0 (NA) | 3 (37.5) | 7 (43.8) | 1 (20.0) | 3 (75.0) | 14 (42.4) |
| *PPM1D* | Control | 0 (NA) | 0 (0.0) | 0 (0.0) | 0 (0.0) | 0 (0.0) | 0 (0.0) |
|  | Low | 0 (NA) | 0 (0.0) | 0 (0.0) | 0 (0.0) | 0 (0.0) | 0 (0.0) |
|  | High | 0 (NA) | 3 (100.0) | 3 (100.0) | 2 (100.0) | 2 (100.0) | 10 (100.0) |
| *TP53* | Control | 0 (NA) | 0 (NA) | 1 (100.0) | 0 (0.0) | 0 (NA) | 1 (50.0) |
|  | Low | 0 (NA) | 0 (NA) | 0 (0.0) | 0 (0.0) | 0 (NA) | 0 (0.0) |
|  | High | 0 (NA) | 0 (NA) | 0 (0.0) | 1 (100.0) | 0 (NA) | 1 (50.0) |
| *ASXL1* | Control | 0 (NA) | 1 (50.0) | 0 (NA) | 0 (0.0) | 0 (0.0) | 1 (25.0) |
|  | Low | 0 (NA) | 1 (50.0) | 0 (NA) | 0 (0.0) | 0 (0.0) | 1 (25.0) |
|  | High | 0 (NA) | 0 (0.0) | 0 (NA) | 1 (100.0) | 1 (100.0) | 2 (50.0) |
| *CBL* | Control | 0 (NA) | 0 (0.0) | 0 (NA) | 1 (100.0) | 0 (0.0) | 1 (33.3) |
|  | Low | 0 (NA) | 1 (100.0) | 0 (NA) | 0 (0.0) | 0 (0.0) | 1 (33.3) |
|  | High | 0 (NA) | 0 (0.0) | 0 (NA) | 0 (0.0) | 1 (100.0) | 1 (33.3) |
| *RAD21* | Control | 0 (NA) | 0 (0.0) | 0 (NA) | 0 (NA) | 0 (0.0) | 0 (0.0) |
|  | Low | 0 (NA) | 0 (0.0) | 0 (NA) | 0 (NA) | 0 (0.0) | 0 (0.0) |
|  | High | 0 (NA) | 1 (100.0) | 0 (NA) | 0 (NA) | 2 (100.0) | 3 (100.0) |
| *ATM* | Control | 0 (NA) | 0 (0.0) | 1 (50.0) | 1 (100.0) | 0 (NA) | 2 (50.0) |
|  | Low | 0 (NA) | 1 (100.0) | 0 (0.0) | 0 (0.0) | 0 (NA) | 1 (25.0) |
|  | High | 0 (NA) | 0 (0.0) | 1 (50.0) | 0 (0.0) | 0 (NA) | 1 (25.0) |
| *FBXW7* | Control | 0 (NA) | 2 (100.0) | 0 (NA) | 0 (0.0) | 0 (NA) | 2 (66.7) |
|  | Low | 0 (NA) | 0 (0.0) | 0 (NA) | 0 (0.0) | 0 (NA) | 0 (0.0) |
|  | High | 0 (NA) | 0 (0.0) | 0 (NA) | 1 (100.0) | 0 (NA) | 1 (33.3) |
| *GNAS* | Control | 0 (NA) | 3 (60.0) | 0 (0.0) | 0 (0.0) | 0 (NA) | 3 (42.9) |
|  | Low | 0 (NA) | 0 (0.0) | 0 (0.0) | 1 (100.0) | 0 (NA) | 1 (14.3) |
|  | High | 0 (NA) | 2 (40.0) | 1 (100.0) | 0 (0.0) | 0 (NA) | 3 (42.9) |
| *GNB1* | Control | 0 (NA) | 0 (NA) | 0 (NA) | 0 (0.0) | 0 (NA) | 0 (0.0) |
|  | Low | 0 (NA) | 0 (NA) | 0 (NA) | 1 (100.0) | 0 (NA) | 1 (100.0) |
|  | High | 0 (NA) | 0 (NA) | 0 (NA) | 0 (0.0) | 0 (NA) | 0 (0.0) |
| *RUNX1* | Control | 0 (NA) | 3 (27.3) | 4 (57.1) | 1 (100.0) | 0 (NA) | 8 (42.1) |
|  | Low | 0 (NA) | 7 (63.6) | 3 (42.9) | 0 (0.0) | 0 (NA) | 10 (52.6) |
|  | High | 0 (NA) | 1 (9.1) | 0 (0.0) | 0 (0.0) | 0 (NA) | 1 (5.3) |
| *SH2B3* | Control | 0 (NA) | 3 (60.0) | 0 (0.0) | 0 (0.0) | 0 (NA) | 3 (42.9) |
|  | Low | 0 (NA) | 1 (20.0) | 1 (100.0) | 1 (100.0) | 0 (NA) | 3 (42.9) |
|  | High | 0 (NA) | 1 (20.0) | 0 (0.0) | 0 (0.0) | 0 (NA) | 1 (14.3) |

Values are presented as count (percentage); “NA” indicates not applicable.

Abbreviations: RAIT (radioactive iodine therapy); VAF (variant allele frequency)

**Table S6.** Overview of CH mutations of patients with paired samples before and after RAIT.

| **No.** | **Age_CH_**  **before**  **RAIT**  **(years)** | **Days between blood**  **draw** | **RAIT**  **dose**  **(GBq)** | | **Changes in the number of CH mutations** | | | | **Sampling**  **timing**  **from**  **RAIT** | | **CH mutations with maximum VAF** | | | | | | **Hematologic parameters** | | | | | | | | | | | | | | | | | | | | | | | |
| --- | --- | --- | --- | --- | --- | --- | --- | --- | --- | --- | --- | --- | --- | --- | --- | --- | --- | --- | --- | --- | --- | --- | --- | --- | --- | --- | --- | --- | --- | --- | --- | --- | --- | --- | --- | --- | --- | --- | --- | --- |
|  |  |  |  |  | **Emerged/**  **Increased** | | | **Vanished/**  **Decreased** |  |  | **Gene** | | **Protein**  **change** | | **VAF (%)** | | **WBC (10^9^/L)** | | **RBC (10^12^/L)** | | **Hb (g/dL)** | | **HCT (%)** | | **MCV (fL)** | | **MCH (pg)** | | **MCHC (g/dL)** | | **RDW (%)** | | **PLT (10^9^/L)** | | **PCT (%)** | | **MPV (fL)** | | **PDW (%)** | |
| #1 | 46.2 | 421 | | 3.7 | | 2 | 5 | | | Pre | | *DNMT3A* | | Splicing | | 0.6 | | 18.13 | | 4.13 | | 12.2 | | 37 | | 89.6 | | 29.5 | | 33 | | 13.1 | | 302 | | 0.31 | | 10.1 | | 11.2 |
|  |  |  |  |  |  |  |  |  |  | Post | | *STAT5B* | | p.R241W | | 1.3 | | 4.08 | | 4.73 | | 13.6 | | 40.8 | | 86.3 | | 28.8 | | 33.3 | | 12.7 | | 233 | | 0.24 | | 10.4 | | 11.4 |
| #2 | 35.8 | 476 | | 1.85 | | 4 | 0 | | | Pre | | *GATA2* | | p.A194G | | 0.6 | | 8.17 | | 5.2 | | 14.8 | | 42.6 | | 81.9 | | 28.5 | | 34.7 | | 13.9 | | 378 | | 0.36 | | 9.4 | | 11.1 |
|  |  |  |  |  |  |  |  |  |  | Post | | *GATA2* | | p.A194G | | 0.7 | | 9.28 | | 5.01 | | 14.6 | | 41.7 | | 83.2 | | 29.1 | | 35 | | 12.5 | | 436 | | 0.41 | | 9.3 | | 10 |
| #3 | 40.1 | 496 | | 1.85 | | 8 | 7 | | | Pre | | *STAT3* | | p.R729fs | | 0.7 | | 5.54 | | 4.77 | | 13.4 | | 41.2 | | 86.4 | | 28.1 | | 32.5 | | 11.7 | | 285 | | 0.3 | | 10.6 | | 13.2 |
|  |  |  |  |  |  |  |  |  |  | Post | | *CNOT3* | | p.I270L | | 1.0 | | 6.11 | | 4.28 | | 12.1 | | 36.4 | | 85 | | 28.3 | | 33.2 | | 12.6 | | 218 | | 0.2 | | 9.3 | | 10.2 |
| #4 | 54.6 | 1562 | | 3.7 | | 6 | 12 | | | Pre | | *NOTCH1* | | p.R56* | | 0.6 | | 7.14 | | 4.22 | | 12 | | 36 | | 85.3 | | 28.4 | | 33.3 | | 12.6 | | 238 | | 0.22 | | 9.4 | | 10.7 |
|  |  |  |  |  |  |  |  |  |  | Post | | *STAT5B* | | p.E269fs | | 0.7 | | 11.02 | | 3.91 | | 11.3 | | 33.1 | | 84.7 | | 28.9 | | 34.1 | | 12.7 | | 244 | | 0.24 | | 9.9 | | 10.4 |
| #5 | 32.3 | 487 | | 1.85 | | 4 | 10 | | | Pre | | *STAG2* | | p.A631V | | 0.7 | | 9.03 | | 5.97 | | 17.6 | | 51.5 | | 86.3 | | 29.5 | | 34.2 | | 12.3 | | 281 | | 0.28 | | 10 | | 12.8 |
|  |  |  |  |  |  |  |  |  |  | Post | | *MED12* | | p.A1775T | | 0.8 | | 7.99 | | 5.98 | | 17.6 | | 50.8 | | 84.9 | | 29.4 | | 34.6 | | 12.8 | | 270 | | 0.28 | | 10.5 | | 11.9 |
| #6 | 61.6 | 478 | | 1.85 | | 5 | 8 | | | Pre | | *DNMT3A* | | p.QAA573del | | 1.0 | | 9.37 | | 4.65 | | 14.1 | | 42.6 | | 91.6 | | 30.3 | | 33.1 | | 11.9 | | 372 | | 0.35 | | 9.4 | | 9.9 |
|  |  |  |  |  |  |  |  |  |  | Post | | *GNAS* | | p.A210S | | 2.1 | | 7.43 | | 4.68 | | 14.6 | | 44 | | 94 | | 31.2 | | 33.2 | | 11.8 | | 266 | | 0.25 | | 9.6 | | 10.3 |
| #7 | 56 | 574 | | 2.22 | | 4 | 9 | | | Pre | | *SMARCA2* | | p.Q532* | | 0.9 | | 5.6 | | 4.42 | | 13.5 | | 39 | | 88.2 | | 30.5 | | 34.6 | | 11.8 | | 211 | | 0.23 | | 11.1 | | 13.3 |
|  |  |  |  |  |  |  |  |  |  | Post | | *KMT2A* | | p.R2811H | | 0.6 | | 4.64 | | 3.95 | | 12.6 | | 36.4 | | 92.2 | | 31.9 | | 34.6 | | 12.2 | | 164 | | 0.18 | | 10.9 | | 13.2 |
| #8 | 52.9 | 640 | | 2.22 | | 1 | 6 | | | Pre | | *DNMT3A* | | p.V657M | | 0.7 | | 5.53 | | 4.18 | | 11.9 | | 37.5 | | 89.7 | | 28.5 | | 31.7 | | 13.2 | | 246 | | 0.26 | | 10.6 | | 12.1 |
|  |  |  |  |  |  |  |  |  |  | Post | | *RUNX1* | | p.E422A | | 0.7 | | 5.24 | | 4.2 | | 12 | | 37.6 | | 89.5 | | 28.6 | | 31.9 | | 13.2 | | 205 | | 0.21 | | 10.2 | | 11.1 |
| #9 | 41.9 | 563 | | 2.22 | | 3 | 4 | | | Pre | | *U2AF1* | | p.S231L | | 0.9 | | 7.35 | | 3.95 | | 11.8 | | 35.4 | | 89.6 | | 29.9 | | 33.3 | | 12.2 | | 368 | | 0.35 | | 9.5 | | 10.1 |
|  |  |  |  |  |  |  |  |  |  | Post | | *EP300* | | p.P2292fs | | 0.8 | | 8.76 | | 4.17 | | 13 | | 38.8 | | 93 | | 31.2 | | 33.5 | | 12.4 | | 332 | | 0.3 | | 9.1 | | 9.6 |
| #10 | 43.7 | 539 | | 2.22 | | 5 | 9 | | | Pre | | *STAT5B* | | p.Q260* | | 0.8 | | 4.46 | | 3.97 | | 8.3 | | 27.8 | | 70 | | 20.9 | | 29.9 | | 17.2 | | 372 | | 0.37 | | 10 | | 10.4 |
|  |  |  |  |  |  |  |  |  |  | Post | | *STAT3* | | p.R729fs | | 0.9 | | 4.73 | | 4.09 | | 9.2 | | 31 | | 75.8 | | 22.5 | | 29.7 | | 19.7 | | 279 | | 0.27 | | 9.8 | | 10.9 |
| #11 | 56.7 | 567 | | 2.22 | | 9 | 15 | | | Pre | | *SMARCA2* | | p.K492N | | 0.8 | | 6.99 | | 5.27 | | 14.6 | | 43.3 | | 82.2 | | 27.7 | | 33.7 | | 11.9 | | 301 | | 0.33 | | 10.9 | | 13.4 |
|  |  |  |  |  |  |  |  |  |  | Post | | *U2AF1* | | p.S231L | | 0.7 | | 9.81 | | 4.83 | | 13.7 | | 39.8 | | 82.4 | | 28.4 | | 34.4 | | 12 | | 261 | | 0.27 | | 10.3 | | 12.3 |
| #12 | 55.7 | 515 | | 2.22 | | 12 | 20 | | | Pre | | *GNAS* | | p.A259V | | 1.6 | | 6.34 | | 4.31 | | 13 | | 40.2 | | 93.3 | | 30.2 | | 32.3 | | 12.7 | | 348 | | 0.32 | | 9.2 | | 9.8 |
|  |  |  |  |  |  |  |  |  |  | Post | | *CHD4* | | p.C475Y | | 0.8 | | 6.48 | | 4.78 | | 14.1 | | 43.9 | | 91.8 | | 29.5 | | 32.1 | | 12.1 | | 357 | | 0.32 | | 8.8 | | 9.3 |
| #13 | 53.6 | 570 | | 2.22 | | 4 | 8 | | | Pre | | *CNOT3* | | p.A330T | | 0.5 | | 5.18 | | 4.52 | | 12.5 | | 38.1 | | 84.3 | | 27.7 | | 32.8 | | 12.5 | | 281 | | 0.31 | | 10.9 | | 12.6 |
|  |  |  |  |  |  |  |  |  |  | Post | | *JAK3* | | p.A746T | | 0.7 | | 7.86 | | 4.91 | | 14.2 | | 42 | | 85.5 | | 28.9 | | 33.8 | | 11.8 | | 308 | | 0.32 | | 10.5 | | 12.1 |
| #14 | 43.4 | 350 | | 1.85 | | 3 | 19 | | | Pre | | *SETBP1* | | p.A1561fs | | 1.4 | | 8.41 | | 4.1 | | 12.7 | | 37.9 | | 92.4 | | 31 | | 33.5 | | 13 | | 198 | | 0.22 | | 11 | | 12.8 |
|  |  |  |  |  |  |  |  |  |  | Post | | *SETBP1* | | p.A1561fs | | 0.7 | | 4.5 | | 4.5 | | 14 | | 41.5 | | 92.2 | | 31.1 | | 33.7 | | 12.4 | | 190 | | 0.22 | | 11.4 | | 13.9 |
| #15 | 48.4 | 538 | | 2.22 | | 10 | 20 | | | Pre | | *TET2* | | p.S1050* | | 0.9 | | 7.39 | | 5.1 | | 13.4 | | 40 | | 78.4 | | 26.3 | | 33.5 | | 13.9 | | 409 | | 0.44 | | 10.9 | | 13 |
|  |  |  |  |  |  |  |  |  |  | Post | | *TET2* | | p.S1050* | | 1.1 | | 8.12 | | 4.74 | | 12.1 | | 37.2 | | 78.5 | | 25.5 | | 32.5 | | 14 | | 336 | | 0.35 | | 10.5 | | 11.8 |
| #16 | 43.8 | 634 | | 2.22 | | 7 | 8 | | | Pre | | *RUNX1* | | p.A440V | | 1.0 | | 5.6 | | 4.44 | | 13.7 | | 40.3 | | 90.8 | | 30.9 | | 34 | | 12.4 | | 300 | | 0.33 | | 11.1 | | 13.4 |
|  |  |  |  |  |  |  |  |  |  | Post | | *TET2* | | p.G1288D | | 1.0 | | 6.76 | | 4.29 | | 13 | | 39.7 | | 92.5 | | 30.3 | | 32.7 | | 11.9 | | 298 | | 0.32 | | 10.9 | | 12.5 |
| #17 | 54.2 | 626 | | 2.22 | | 5 | 6 | | | Pre | | *U2AF1* | | p.S231L | | 1.0 | | 5.1 | | 4.35 | | 12.9 | | 39.3 | | 90.2 | | 29.7 | | 32.9 | | 12.4 | | 199 | | 0.17 | | 8.6 | | 13.1 |
|  |  |  |  |  |  |  |  |  |  | Post | | *CUX1* | | p.C628Y | | 0.6 | | 6.9 | | 4.57 | | 13.7 | | 39.4 | | 86.2 | | 30 | | 34.8 | | 11.9 | | 234 | | 0.25 | | 10.7 | | 12.6 |
| #18 | 52.6 | 728 | | 2.22 | | 1 | 9 | | | Pre | | *DNM2* | | p.A756T | | 0.9 | | 4.21 | | 4.02 | | 12.4 | | 36.9 | | 91.8 | | 30.8 | | 33.6 | | 11.7 | | 247 | | 0.26 | | 10.6 | | 12 |
|  |  |  |  |  |  |  |  |  |  | Post | | *PDGFRA* | | p.A518T | | 0.5 | | 4.2 | | 4.19 | | 12.7 | | 37.9 | | 90.5 | | 30.3 | | 33.5 | | 11.9 | | 210 | | 0.24 | | 11.6 | | 13.2 |
| #19 | 50.4 | 574 | | 1.11 | | 8 | 14 | | | Pre | | *GIGYF2* | | p.F147fs | | 0.8 | | 3.67 | | 4.28 | | 12.9 | | 39.7 | | 92.8 | | 30.1 | | 32.5 | | 12.2 | | 166 | | 0.18 | | 10.9 | | 13.2 |
|  |  |  |  |  |  |  |  |  |  | Post | | *JAK3* | | p.A1090T | | 0.7 | | 4.7 | | 4.17 | | 13.6 | | 40.6 | | 97.4 | | 32.6 | | 33.5 | | 11.8 | | 145 | | 0.15 | | 10.5 | | 12.3 |
| #20 | 33.6 | 1778 | | 6.66 | | 7 | 11 | | | Pre | | *PDGFRA* | | p.D1033V | | 3.1 | | 5.1 | | 3.6 | | 8.5 | | 26.5 | | 73.6 | | 23.5 | | 32 | | 17.2 | | 259 | | 0.25 | | 9.8 | | 11 |
|  |  |  |  |  |  |  |  |  |  | Post | | *SH2B3* | | p.E267D | | 1.0 | | 4.67 | | 4.59 | | 10.4 | | 34 | | 74.1 | | 22.7 | | 30.6 | | 16.6 | | 336 | | 0.33 | | 9.8 | | 10.4 |
| #21 | 42.5 | 329 | | 1.85 | | 5 | 10 | | | Pre | | *MED12* | | p.E810fs | | 0.7 | | 5.72 | | 4.4 | | 13.2 | | 39.7 | | 90.2 | | 30 | | 33.2 | | 11.7 | | 236 | | 0.25 | | 10.8 | | 12.8 |
|  |  |  |  |  |  |  |  |  |  | Post | | *KIT* | | p.A755T | | 0.7 | | 11.4 | | 4.09 | | 12.5 | | 36.1 | | 88.3 | | 30.6 | | 34.6 | | 11.7 | | 250 | | 0.26 | | 10.3 | | 11.5 |
| #22 | 28.9 | 343 | | 1.85 | | 3 | 7 | | | Pre | | *DNMT3A* | | p.I310S | | 1.4 | | 4.55 | | 4.56 | | 13.4 | | 40 | | 87.7 | | 29.4 | | 33.5 | | 13 | | 346 | | 0.33 | | 9.5 | | 10 |
|  |  |  |  |  |  |  |  |  |  | Post | | *U2AF1* | | p.S231L | | 0.9 | | 7.47 | | 4.6 | | 13.6 | | 40.7 | | 88.5 | | 29.6 | | 33.4 | | 12.8 | | 310 | | 0.28 | | 9.1 | | 9.6# |
| #23 | 62.9 | 739 | | 2.22 | | 5 | 5 | | | Pre | | *TET2* | | p.Q969fs | | 1.1 | | 6.66 | | 3.79 | | 12 | | 34.5 | | 91 | | 31.7 | | 34.8 | | 12.7 | | 146 | | 0.15 | | 10.6 | | 12.6 |
|  |  |  |  |  |  |  |  |  |  | Post | | *TET2* | | p.Q969fs | | 1.5 | | 6.07 | | 4.2 | | 12.9 | | 39.3 | | 93.6 | | 30.7 | | 32.8 | | 13.2 | | 139 | | 0.15 | | 10.7 | | 12.5 |
| #24 | 21.1 | 1467 | | 37 | | 0 | 12 | | | Pre | | *KDM6A* | | p.A1252V | | 1.1 | | 16.9 | | 4.66 | | 13.7 | | 40 | | 85.8 | | 29.4 | | 34.3 | | 13.2 | | 224 | | 0.19 | | 8.5 | | 8.7 |
|  |  |  |  |  |  |  |  |  |  | Post | | Not detected | | | | | | 4.8 | | 5.03 | | 15 | | 44.9 | | 89.3 | | 29.8 | | 33.4 | | 12.9 | | 275 | | 0.24 | | 8.6 | | 8.6 |

Abbreviations: RAIT (radioactive iodine therapy); Age_CH_ (age at blood draw for clonal hematopoiesis test); Age_RAIT_ (age at the first session of RAIT); VAF (variant allele frequency); CH (clonal hematopoiesis); BMI (body mass index); WBC (white blood cells); RBC (red blood cells); Hb (hemoglobin); HCT (hematocrit); MCV (mean corpuscular volume); MCH (mean corpuscular hemoglobin); MCHC (mean corpuscular hemoglobin concentration); RDW (red cell distribution width); PLT (platelet); MPV (mean platelet volume); PDW (platelet distribution width)
